# Towards Elucidating Carnosic Acid Biosynthesis in *Lamiaceae*: Functional Characterization of the Three First Steps of the Pathway in *Salvia fruticosa* and *Rosmarinus officinalis*

**DOI:** 10.1371/journal.pone.0124106

**Published:** 2015-05-28

**Authors:** Dragana Božić, Dimitra Papaefthimiou, Kathleen Brückner, Ric C. H. de Vos, Constantinos A. Tsoleridis, Dimitra Katsarou, Antigoni Papanikolaou, Irini Pateraki, Fani M. Chatzopoulou, Eleni Dimitriadou, Stefanos Kostas, David Manzano, Ulschan Scheler, Albert Ferrer, Alain Tissier, Antonios M. Makris, Sotirios C. Kampranis, Angelos K. Kanellis

**Affiliations:** 1 Group of Biotechnology of Pharmaceutical Plants, Laboratory of Pharmacognosy, Department of Pharmaceutical Sciences, Aristotle University of Thessaloniki, 541 24 Thessaloniki, Greece; 2 Leibniz Institute of Plant Biochemistry, Department of Cell and Metabolic Biology, Halle (Saale), Germany; 3 Plant Research International, Wageningen University and Research Centre, The Netherlands; 4 Laboratory of Organic Chemistry, Department of Chemistry, Aristotle University of Thessaloniki, 541 24 Thessaloniki, Greece; 5 Department of Molecular Genetics, Centre for Research in Agricultural Genomics (CSIC-IRTA-UAB-UB), Bellaterra-Cerdanyola del Vallés, 08193 Barcelona, Spain; 6 Department of Biochemistry and Molecular Biology, Faculty of Pharmacy, University of Barcelona, 08028 Barcelona, Spain; 7 Laboratory of Floriculture, School of Agriculture, Aristotle University of Thessaloniki, 541 24 Thessaloniki, Greece; 8 Institute of Applied Biosciences, Centre for Research and Technology Hellas, Thermi Thessaloniki, Greece; 9 Department of Biochemistry, School of Medicine, University of Crete, P.O. Box 2208, 710 03 Heraklion, Greece; 10 Netherlands Metabolomics Centre, Leiden, The Netherlands; University of Copenhagen, DENMARK

## Abstract

Carnosic acid (CA) is a phenolic diterpene with anti-tumour, anti-diabetic, antibacterial and neuroprotective properties that is produced by a number of species from several genera of the *Lamiaceae* family, including *Salvia fruticosa* (Cretan sage) and *Rosmarinus officinalis* (Rosemary). To elucidate CA biosynthesis, glandular trichome transcriptome data of *S*. *fruticosa* were mined for terpene synthase genes. Two putative diterpene synthase genes, namely *SfCPS *and *SfKSL*, showing similarities to copalyl diphosphate synthase and kaurene synthase-like genes, respectively, were isolated and functionally characterized. Recombinant expression in *Escherichia coli* followed by *in vitro* enzyme activity assays confirmed that SfCPS is a copalyl diphosphate synthase. Coupling of SfCPS with SfKSL, both *in vitro* and in yeast, resulted in the synthesis miltiradiene, as confirmed by 1D and 2D NMR analyses (^1^H, ^13^C, DEPT, COSY H-H, HMQC and HMBC). Coupled transient *in vivo* assays of *SfCPS* and *SfKSL* in *Nicotiana benthamiana* further confirmed production of miltiradiene *in planta*. To elucidate the subsequent biosynthetic step, RNA-Seq data of *S*. *fruticosa* and *R*. *officinalis* were searched for cytochrome P450 (CYP) encoding genes potentially involved in the synthesis of the first phenolic compound in the CA pathway, ferruginol. Three candidate genes were selected, *SfFS*, *RoFS1* and *RoFS2*. Using yeast and *N*. *benthamiana* expression systems, all three where confirmed to be coding for ferruginol synthases, thus revealing the enzymatic activities responsible for the first three steps leading to CA in two *Lamiaceae* genera.

## Introduction

**Fig 1:**
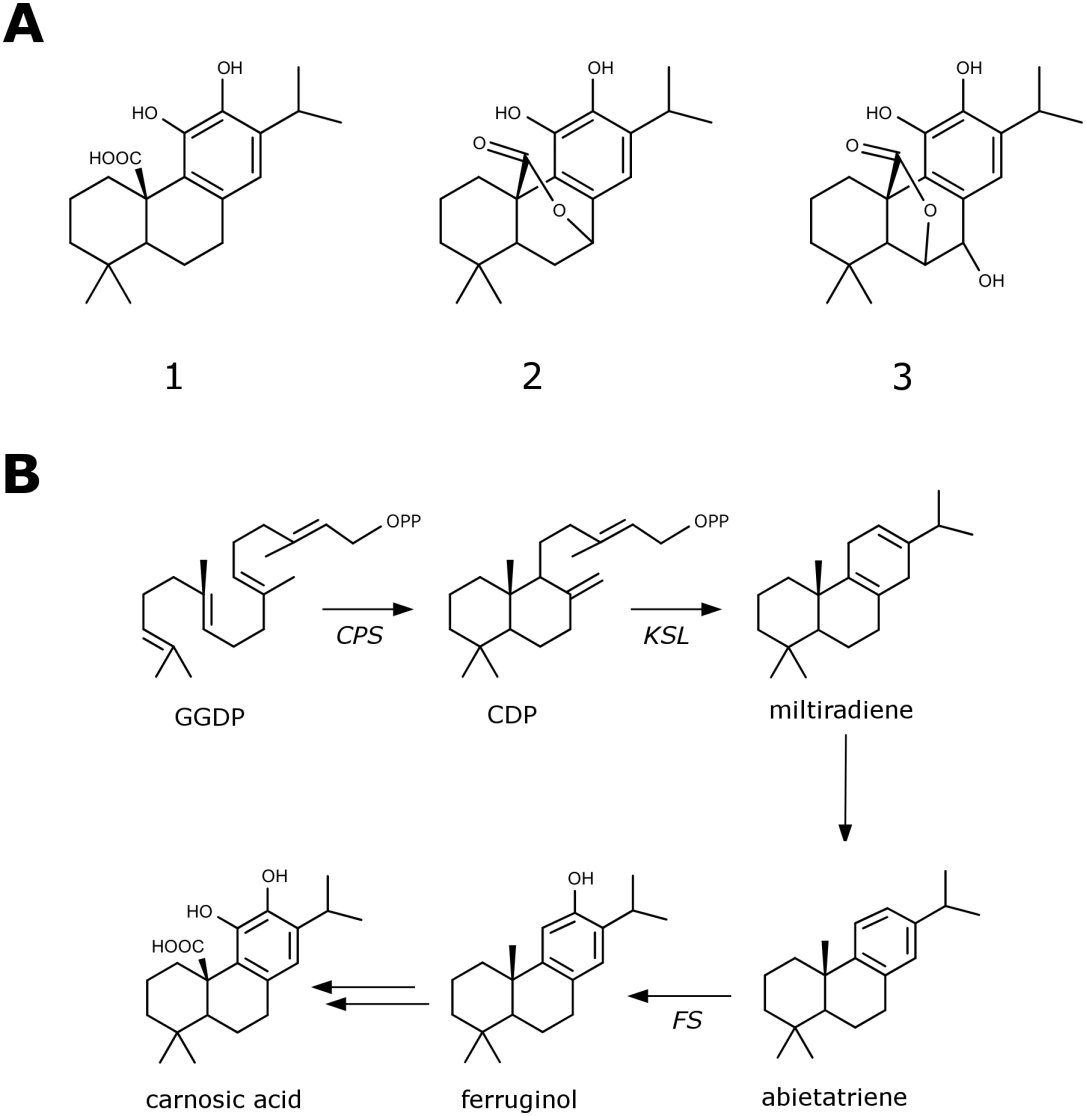
Structure and biosynthesis of labdane-type diterpenes. A) Chemical structure of three phenolic-type diterpenes from *S*. *fruticosa*. 1) carnosic acid. 2) carnosol. 3) rosmanol. B) The proposed biosynthetic pathway from GGDP to carnosic acid.

Phenolic diterpenes (PDs) belong to a class of labdane-related diterpenes having a phenolic functional group. One of the most studied PDs is carnosic acid (CA). Carnosic acid is of high importance for the food and cosmetic industry, and may also have pharmaceutical applications, due to its strong antioxidant, anti-inflammatory and anticancer properties [1–4]. Diverse biological activities, ranging from neuroprotective [5], antiphotoaging [6], antimicrobial [7], anti-angiogenic [8], hepatoprotective [9], anti-adipogenic [10], anti-hyperglycemic to lipid profile-improving [11] have been reported. Most of these biological activities likely stem from its easily oxidizable *o*-diphenol structure [12]. Due to the significance of CA for the cosmetic, food and pharmaceutical industry, there is a great need to understand the processes leading to its biosynthesis. *Salvia fruticosa* and *Rosmarinus officinalis*, plant species of the Mediterranean basin and members of the *Lamiaceae* family, are known to be rich in PDs, especially CA, carnosol (C) and rosmanol (Fig 1A) [13–17]. Elucidation of the CA biosynthetic pathway in *Lamiaceae*, by isolating and functionally characterizing the genes involved, could lead to the development of novel methods for the biotechnologically sustainable production of this molecule.

It has been proposed that biosynthesis of PDs starts with two successive cyclization steps [18]. The initial cyclization reaction converts the common diterpene precursor, geranylgeranyl diphosphate (GGDP), into the bicyclic copalyl diphosphate (CDP). This protonation-initiated cyclization reaction is catalyzed by class II diterpene synthases (class II diTPSs), termed copalyl diphosphate synthases (CPS), which form various stereoisomers of CDP (e.g. normal, *ent*-, or *syn*-CDP). Products of class II diterpene synthases are further used by specific class I diterpene synthases (class I diTPSs) to form specific polycyclic compounds [19]. Diterpene synthases harbour characteristic aspartate-rich catalytic motifs in their active sites. The class II diterpene synthases contain a conserved DXDD motif involved in the protonation-initiated cyclization of GGDP into bicyclic diphosphates [18,20,21]. The class I diterpene synthases contain a DDXXD motif involved in metal-dependent ionization of the prenyl diphosphate substrates [22,23]. Following the formation of the parent skeleton by the diterpene synthases, cytochrome P450 monooxygenases frequently catalyze secondary transformations of the newly formed polycyclic diterpenes.

Gao et al. (2009) reported the functional characterization of two diterpene synthases forming the precursors of the abietane-type norditerpenoid tanshinones in *Salvia miltiorrhiza*. The class II diterpene cyclase produces the normal stereoisomer of CDP from GGDP and was termed CDP synthase (SmCPS). The class I diterpene cyclase that produces miltiradiene, a potential precursor to tanshinones, was named *ent*-kaurene synthase-like (SmKSL) [18]. Recently, genes with high similarity to *SmCPS* and *SmKSL* were characterized from *R*. *officinalis* and shown to code for the same enzymatic activities [24]. Additionally, cytochrome P450 monooxygenases CYP76AH1 and CYP76AH4 that catalyze the formation of ferruginol, the first phenolic diterpene in the sequence of reactions coming after miltiradiene, have also recently been characterized [25,26]. The enzyme CYP76AH1 from *S*. *miltiorrhiza*, is potentially a part of the tanshinone biosynthetic pathway, while CYP76AH4 likely plays a role in the biosynthesis of CA in rosemary. The substrate for these enzymes is abietatriene, a product of either spontaneously occurring or enzymatically catalyzed miltiradiene aromatization [26].

Here, we describe the isolation of two diterpene synthase genes (*SfCPS* and *SfKSL*) and one cytochrome P450 (*SfFS*) gene from *Salvia fruticosa*, responsible for the formation of the CA precursors CDP, miltiradiene and ferruginol, respectively (Fig 1B). In addition, we report the cloning and functional expression of two *R*. *officinalis* ferruginol synthases (RoFS1 and RoFS2) in yeast and *Nicotiana benthamiana*. Characterization of the genes involved in the initial steps of the CA biosynthesis in *S*. *fruticosa* and *R*. *officinalis* is an essential step for the successful elucidation of the CA biosynthesis pathway in *Lamiaceae*.

## Results

### Chemical analysis of genotypes, leaf developmental stages, and organs of *Salvia fruticosa*

**Fig 2:**
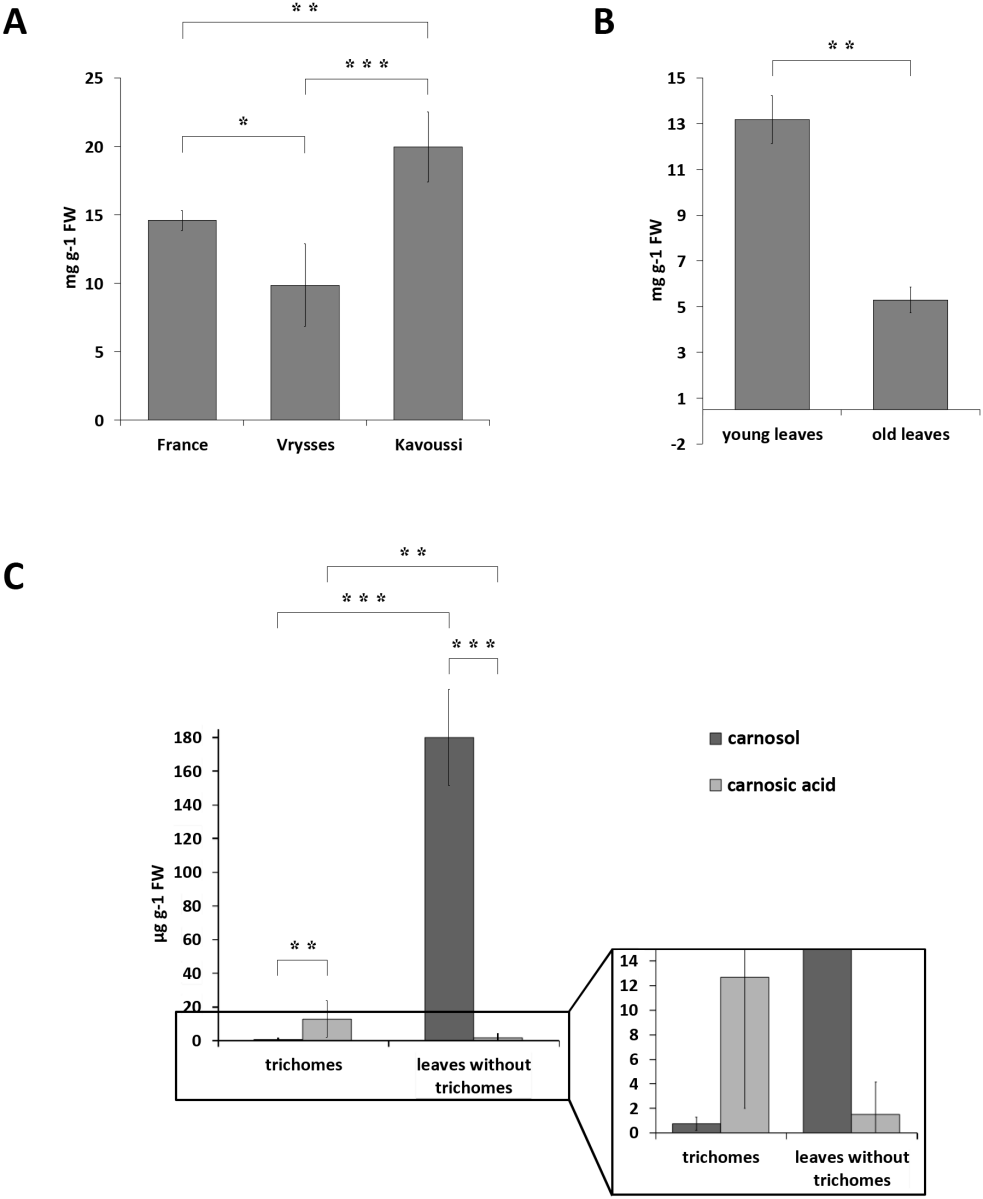
Accumulation of phenolic diterpenes in *S*. *fruticosa* leaves and trichomes. A) Total phenolic diterpenes (PDs) (carnosic acid + carnosol) contents in three populations of *S*. *fruticosa* extracted from whole leaves of all developmental stages. B) Accumulation of total PDs (carnosic acid + carnosol) in young and old leaves of the genotype Kavoussi. C) Carnosic acid and carnosol contents in trichomes and leaves without trichomes of the genotype Kavoussi collected from very young leaves (up to 1cm long). Each bar represents the average of three independent biological samples ± SD. Asterisks denote significant differences between two indicated values (*p < 0.05; **p < 0.01; ***p < 0.001), based on Student’s *t*-test.

To identify the genotype with the highest concentration of PDs, *S*. *fruticosa* plants originating from the Eastern and Western parts of Crete (Greece), namely Kavoussi and Vrysses, respectively, or from a commercial source (France), were grown in the greenhouse and analysed by HPLC with both PDA detector and accurate mass MS for their CA and C contents. This analysis identified the genotype Kavoussi as the richest source in PDs, followed by the commercial and Vrysses genotypes, when whole leaves of all developmental stages were assessed (Fig 2A). Young leaves had significantly higher amounts of the sum of CA and C than old leaves (Fig 2B). In addition, it was found that trichome preparations from young leaves contained higher amounts of CA compared to leaves without trichomes, whereas, surprisingly, C accumulated in high quantities in leaves without trichomes and was present only in trace amounts in isolated trichomes (Fig 2C).

### Isolation and cloning of *SfCPS* and *SfKSL* genes

Previous work provided an EST database from a cDNA library constructed from *S*. *fruticosa* leaf trichome total RNA [27]. Two partial sequences in this database exhibited homology to potential diterpene synthases. The EST contig 195 (824 bp long, consisting of three ESTs, with GeneBank accessions JZ562276, JZ562277 and JZ562278) and contig 66 (706 bp, ESTs with GeneBank accessions JZ562273, JZ562274 and JZ562275), revealed homology to the family of copalyl diphosphate synthases and kaurene synthases, respectively. The two sequences were also identified in another *S*. *fruticosa* leaf trichome EST database (http://www.terpmed.eu/).

**Fig 3:**
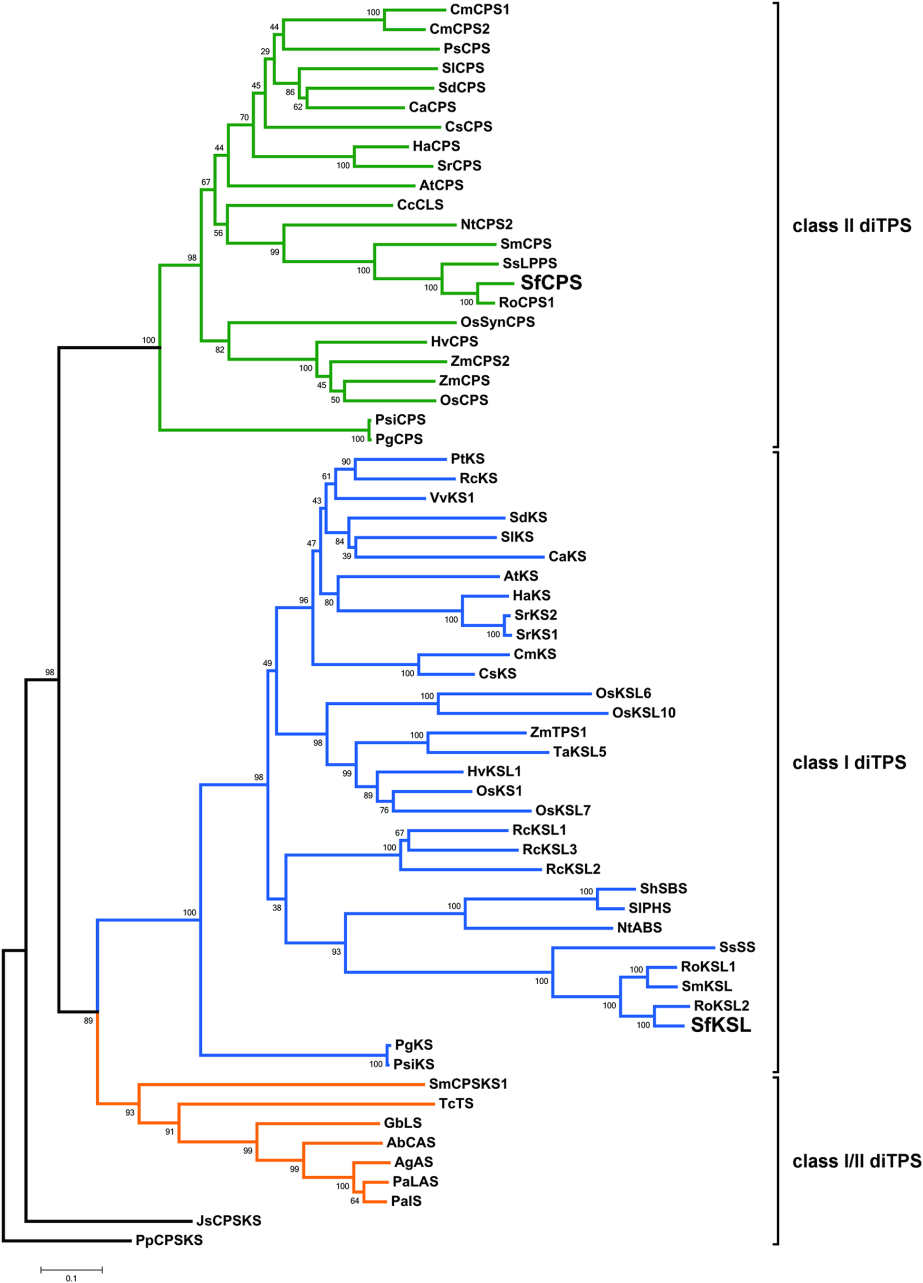
Phylogenetic analysis of SfCPS and SfKSL representing their relatedness to other plant labdane-type diterpenes. The neighbour-joining tree was generated using MEGA version 5 [52] from amino acid sequence alignment. Protein abbreviations and accession numbers are listed in S1 Table. The tree was rooted using *Physcomitrella patens* PpCPS/KS as outgroup.

The entire ORF of both sequences was isolated, partly from the *S*. *fruticosa* trichome cDNA/EST library [27] and partly by RACE-PCR, using *S*. *fruticosa* trichome cDNA as the template. The two diterpene synthases were annotated as SfCPS and SfKSL. The ORFs of *SfCPS* and *SfKSL* consisted of 2391 and 1755 base pairs, respectively. Phylogenetic analysis revealed that SfCPS belongs to the group of CPS proteins, while SfKSL is part of the KSL protein family (Fig 3). Both enzymes belong to the Tps e/f group of terpene synthases [28]. The most similar sequence to the deduced SfCPS amino acid sequence is *Rosmarinus officinalis* copalyl diphosphate synthase—RoCPS1 (88% identity, accession number KF805857). The deduced amino acid sequence of SfKSL showed highest similarity to *Rosmarinus officinalis* kaurene synthase-like 2—RoKSL2 (85% identity, accession number KF805859). Analysis of the isolated sequences with TargetP 1.1 software indicated the existence of putative transit peptides in both sequences, thus suggesting the plastidial localization of the mature proteins. Furthermore, a highly conserved aspartate-rich DxDD motif, characteristic of class II diTPS, which is required for the protonation-dependent cyclization of GGDP, was detected in the SfCPS sequence (S1 Fig). SfKSL, on the other hand, possesses a DDxxD motif, required for Mg^2+^-mediated substrate binding and characteristic of class I diterpene synthases (S2 Fig). SfKSL lacks the γ-domain and therefore likely has an *α/β* bi-domain protein structure.

### Tissue specificity and developmental expression of *SfCPS* and *SfKSL*

**Fig 4:**
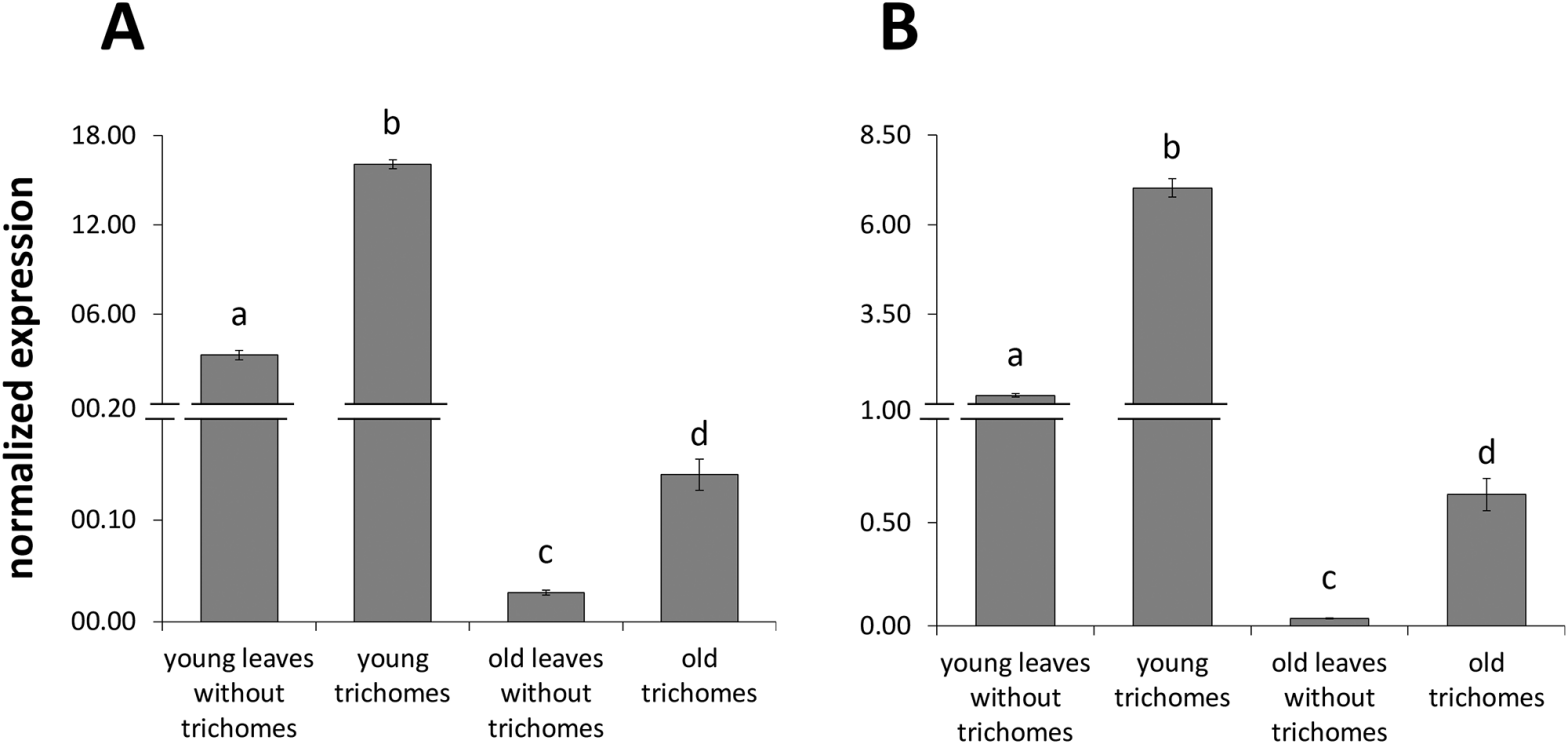
Quantitative expression analysis (qPCR) of *SfCPS* (A) and *SfKSL* (B) in trichomes and leaves without trichomes of young and old leaves. Data bars represent the mean expression levels from three biological replicates ± SE. For each gene, values marked with a different letter are significantly different at p < 0.05, according to Student’s *t*-test. Transcript levels were normalized to *elf4a* gene (endogenous control).

Real-time PCR analysis demonstrated higher expression levels of *SfCPS* and *SfKSL* in isolated trichomes than in leaves without trichomes. Furthermore, a difference in expression levels of these genes could also be observed during leaf development. Young leaves showed higher accumulation of the *SfCPS* and *SfKSL* transcripts, when compared to the fully expanded leaves (Fig 4). This expression pattern was consistent with the trichome and developmental regulation of CA build-up (cf. Figs 2 and 4).

### Miltiradiene is produced in coupled assays with SfCPS and SfKSL

**Fig 5:**
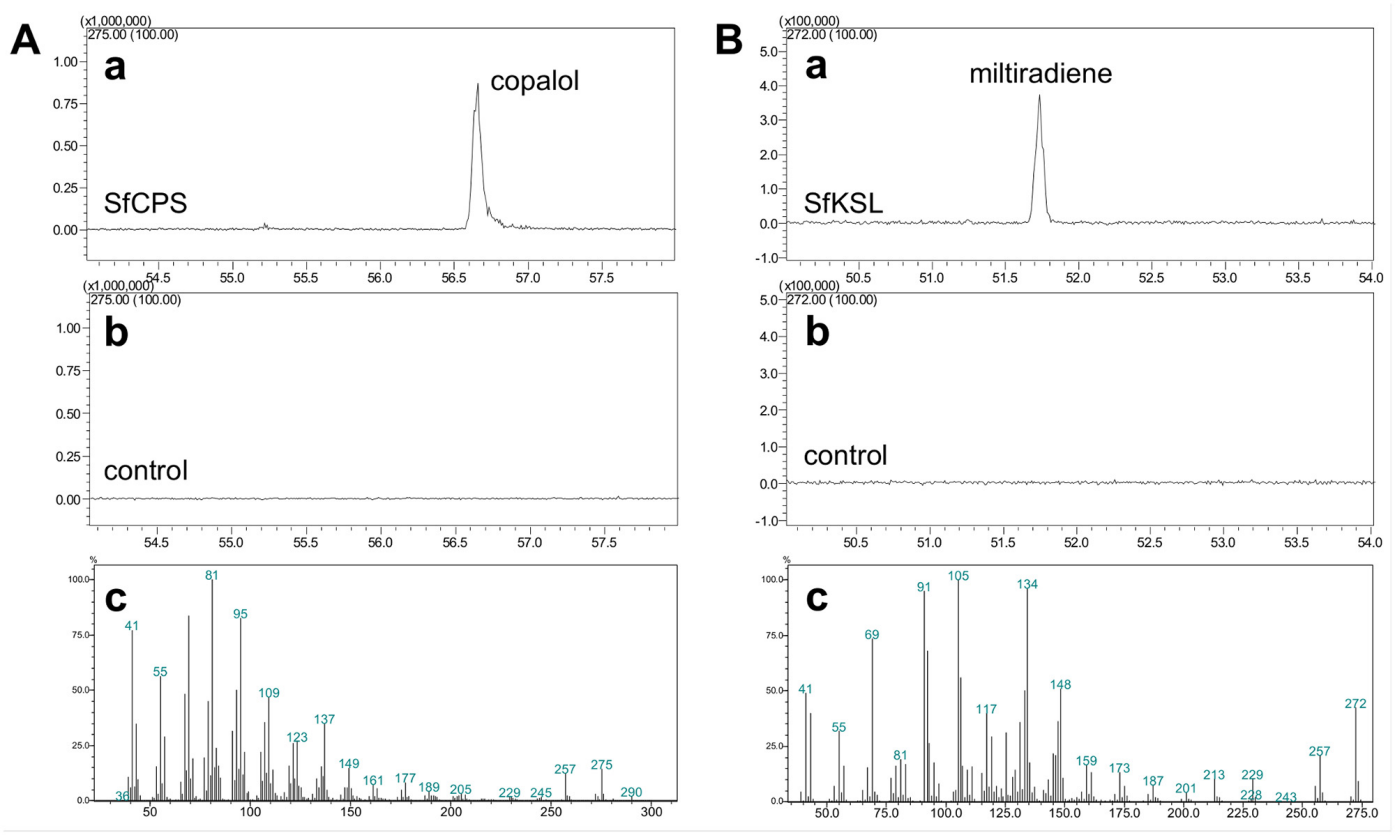
The functional characterization of *SfCPS* and *SfKSL* in *E*. *coli*. A) *SfCPS characterization*. (a) GC-MS profile (275 m/z extracted ion chromatograms) of the dephosphorylated product of mature SfCPS incubated with GGDP. (b) GC-MS of the control reaction- substrate GGDP omitted. (c) Mass spectrum of the product peak- copalol. B) *SfKSL characterization*. (a) GC-MS profile (272 m/z extracted ion chromatograms of the product (miltiradiene) of coupled reaction of SfCPS and SfKSL with GGDP as substrate, b) GC-MS profile of the enzymatic assay with SfCPS omitted. c) Mass spectrum of the reaction product—miltiradiene.

The predicted mature versions of the two diterpene synthases, SfCPS and SfKSL, lacking the putative chloroplast targeting peptides, were expressed in *E*. *coli*. Based on the TargetP 1.1 software prediction, the lengths of the two transit peptides were 30 and 49 amino acids for the SfCPS and SfKSL, respectively. His-tagged SfCPS protein was affinity purified and incubated with GGDP, after which the reaction mixture was subjected to enzymatic hydrolysis by alkaline phosphatase. Hexane-extracted reaction products were analysed by GC-MS. Using this approach, it was possible to identify the dephosphorylated product of the SfCPS catalyzed reaction as copalol, by comparison with the Wiley mass spectra database (Fig 5A).

To functionally characterize the *SfKSL* gene, affinity purified SfKSL protein was assayed *in vitro* simultaneously with SfCPS in order to provide the necessary CDP substrate for the class I-type reaction. GGDP was added to the reaction as the substrate for SfCPS. GC-MS analysis of the hexane-extracted reaction products (Fig 5B) revealed a peak, which most closely corresponded to miltiradiene based on the published mass spectrum [18,24](Fig 5B).

**Fig 6:**
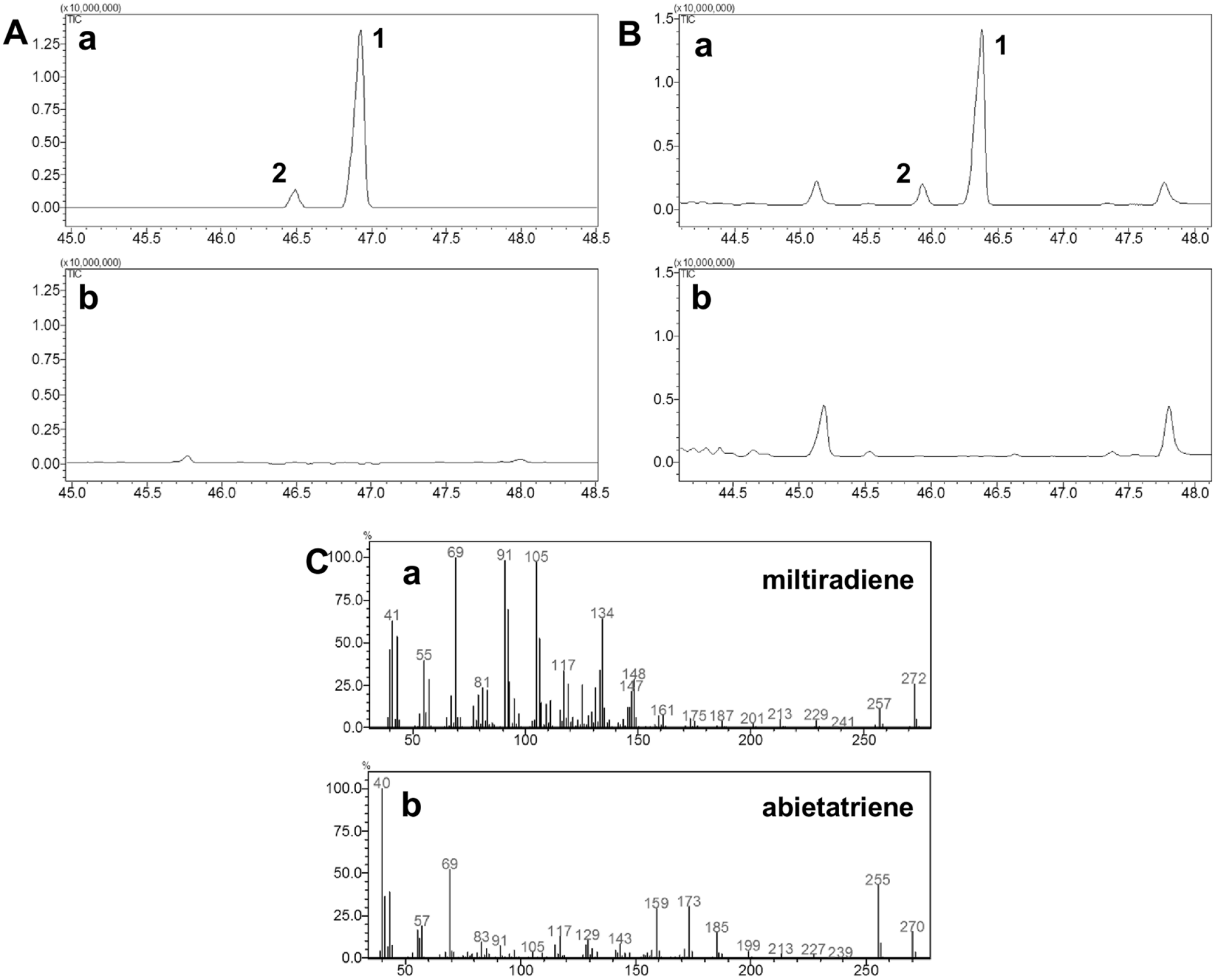
Miltiradiene production in yeast and agro-infiltrated *Nicotiana benthamiana* leaves. A) *Yeast expression assays*. (a) GC-MS profile of a hexane fraction obtained by silica gel flash chromatography containing the purified products (peak 1 and 2) from yeast transformed with *SfCPS* and *SfKSL*. (b) GC-MS profile of non-transformed yeast strain AM104. B) *N*. *benthamiana transient co-expression*: (a) GC-MS profile of *N*. *benthamiana* leaves infiltrated with *SfCPS* and *SfKSL* (showing peaks 1 and 2). (b) GC-MS profile of *N*. *benthamiana* leaves infiltrated with empty vector as a control. C) Mass spectrum for: (a) miltiradiene (peak 1); (b) abietatriene (peak 2).

Next, the two diTPSs enzymatic activities were additionally assayed in yeast cells by recombinant expression and in *N*. *benthamiana* using agroinfiltration. First, *S*. *cerevisiae* strain AM104, which carries a chromosomal integration of *Cistus creticus* GGDP synthase (*CcGGDPS1*) [29], was co-transformed with the two diTPS of interest lacking the predicted transit peptide. Yeast cells extracted with hexane, and silica purified preparations analysed by GC-MS revealed the presence of a miltiradiene-like compound (peak 1, Fig 6A and 6Ca), corroborating the results of the *E*. *coli* assays (compare Figs 5 and 6A). Additionally, the production of another compound (peak 2, Fig 6A) was detected, the mass spectrum of which matched most closely to that of abietatriene (Fig 6Cb), based on comparison with the Wiley mass spectra database.

Secondly, to perform the transient expression *in planta*, binary vector constructs containing the full length *SfCPS* and *SfKSL* ORFs were co-infiltrated into *N*. *benthamiana* leaves, together with a viral silencing suppressor. After 5 days, hexane extracts of infiltrated leaves were subjected to a GC-MS analysis resulting in peaks (Fig 6Ba) and mass spectra (Fig 6Ca and 6Cb) identical to those previously detected in the *E*. *coli* assays and yeast analyses (compare Fig 5, Fig 6A and 6B).

**Fig 7:**
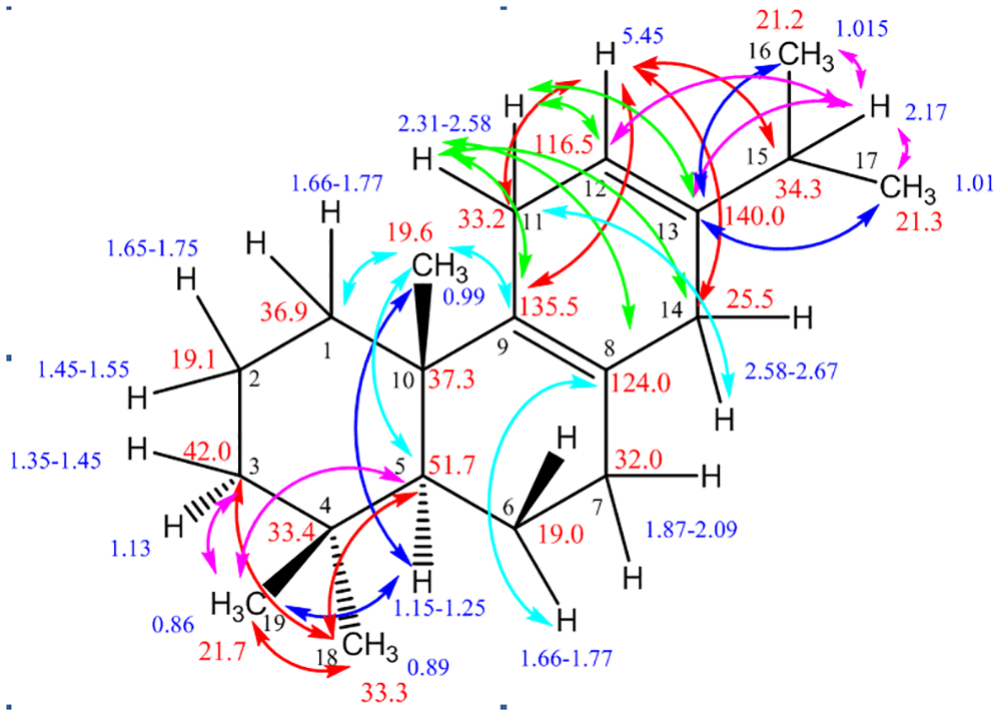
Diagnostic HMBC correlations for miltiradiene. The correlations between carbons and protons via ^*2*^ *J*_*CH*_ and ^*3*^ *J*_*CH*_ couplings are shown. In the case of cyclohexadiene ring correlations are observed also via ^*4*^ *J*_*CH*_ couplings through the double bonds.

Although the mass spectrum of the compound produced by the coupled activity of SfCPS and SfKSL displayed high similarity to the mass spectrum of miltiradiene previously characterized by Gao et al. (2009), many abietane-type diterpenes exhibit highly similar mass spectra. To unambiguously identify this compound, its structure was determined by NMR spectroscopy. For this, a 3.75 l culture of AM104 yeast cells co-expressing the two terpene synthases was grown, yielding approximately 20 mg of the miltiradiene-like compound (Peak 1, Fig 6A and 6Ca), which after silica purification was subjected to NMR analysis (Fig 7). It should be noted that abietatriene also co-eluted in small amounts with the miltiradiene-like molecule, but its presence did not interfere with the structural characterization of the main compound (Peak 2, Fig 6A and 6Cb). On the basis of GC-MS, 1D and 2D NMR data (^1^H, ^13^C, DEPT, COSY H-H, HMQC and HMBC), the purified compound was confirmed as miltiradiene (S4 Fig–S8 Fig).

### Selection, isolation and cloning of *SfFS*, *RoFS1* and *RoFS2*

**Fig 8:**
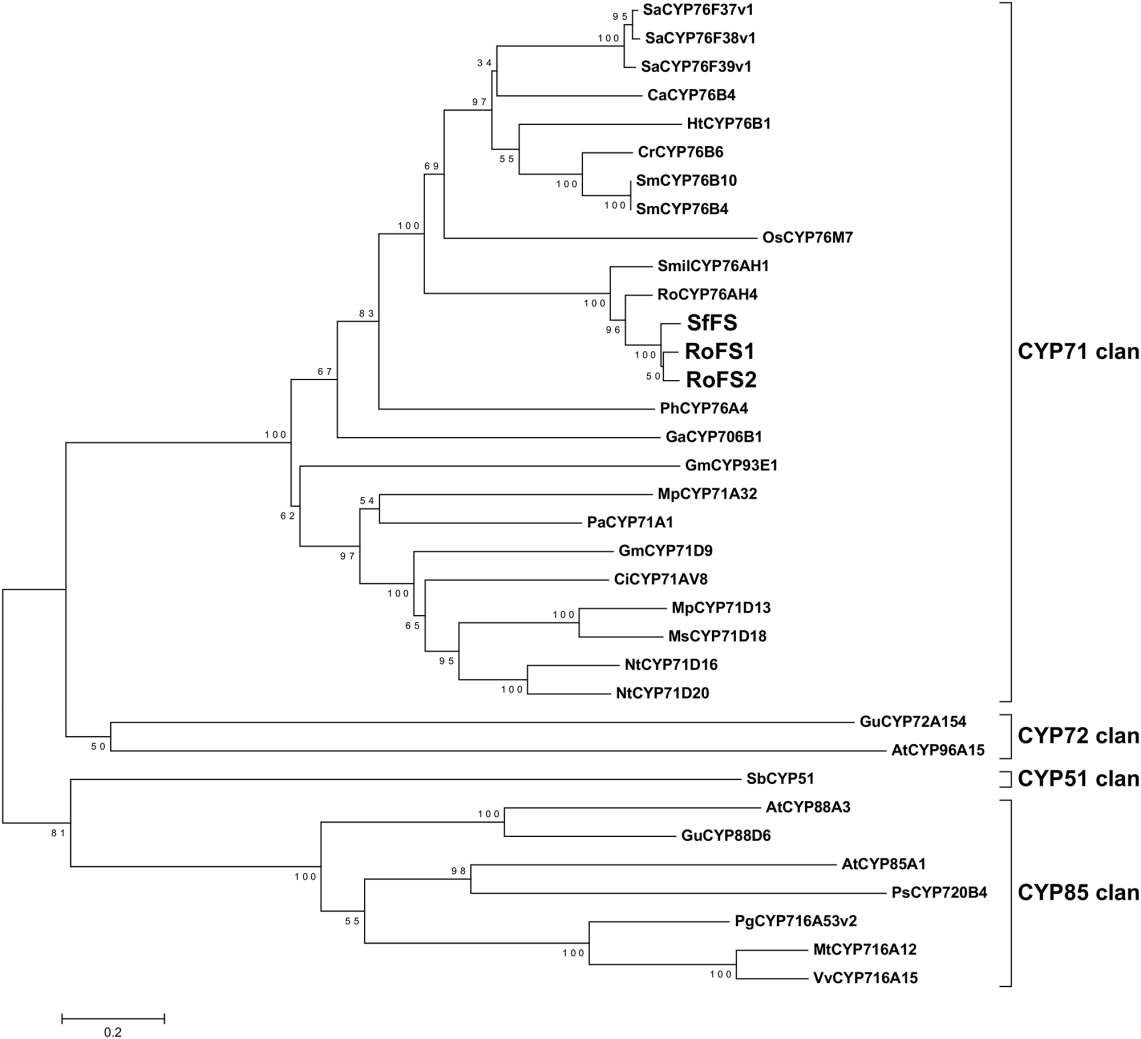
Phylogenetic analysis of SfFS, RoFS1 and RoFS2 representing their relatedness to other plant cytochrome P450s. The neighbour-joining tree was generated using MEGA version 5 [52] from amino acid sequence alignment. Protein abbreviations and accession numbers are listed in S2 Table.

Our previous *S*. *fruticosa* EST analysis [27] gave a limited number of CYPs. In order to identify the P450 proteins responsible for the downstream steps of the CA biosynthetic pathway, it was considered necessary to apply RNA-Seq on newly isolated trichomes from young leaves of *S*. *fruticosa* and *R*. *officinalis* (http://www.terpmed.eu/). These tissues where chosen because they accumulate CA at higher levels compared to the old leaves (see Fig 2) and exhibit a similar pattern of transcript levels of *SfCPS* and *SfKS* (see Fig 4). Contigs encoding full length P450s were selected by BLAST searches. Eighteen candidate sequences from *S*. *fruticosa* and eighteen from *R*. *officinalis* could thus be identified. Among those, two sequences from *S*. *fruticosa* and two from *R*. *officinalis* that shared high similarity at the nucleotide level to the recently described ferruginol synthase genes from *S*. *miltiorrhiza* and *R*. *officinalis* [23, 28] were selected for further study. Upon PCR amplification and sequencing the two *S*. *fruticosa* candidate singletons, *Sfru*.*N01*.*C016156* and *Sfru*.*N01*.*C021415*, turned out to correspond to different regions of the same gene. This gene, together with the two *R*. *officinalis* genes was successfully isolated and sequenced and the three candidate P450 genes were assigned the names *SfFS*, *RoFS1* and *RoFS2* (an alignment of the corresponding protein sequences and the ferruginol synthases from *S*. *miltiorrhiza* and *R*. *officinalis* is given in the S3 Fig). The gene sequences were deposited in the GenBank (accession numbers: *SfFS*: KP091842, *RoFS1*: KP091843, *RoFS2*: KP091844). Detailed phylogenetic analysis of these sequences was performed (Fig 8), indicating that the corresponding P450s belong to the CYP76 subgroup of the large CYP71 clan. CYP71 is a multi-family clan including more than a half of all plant CYPs [30]. Most of the CYP76 P450s are involved in the metabolism of terpenoids [31].

**Fig 9:**
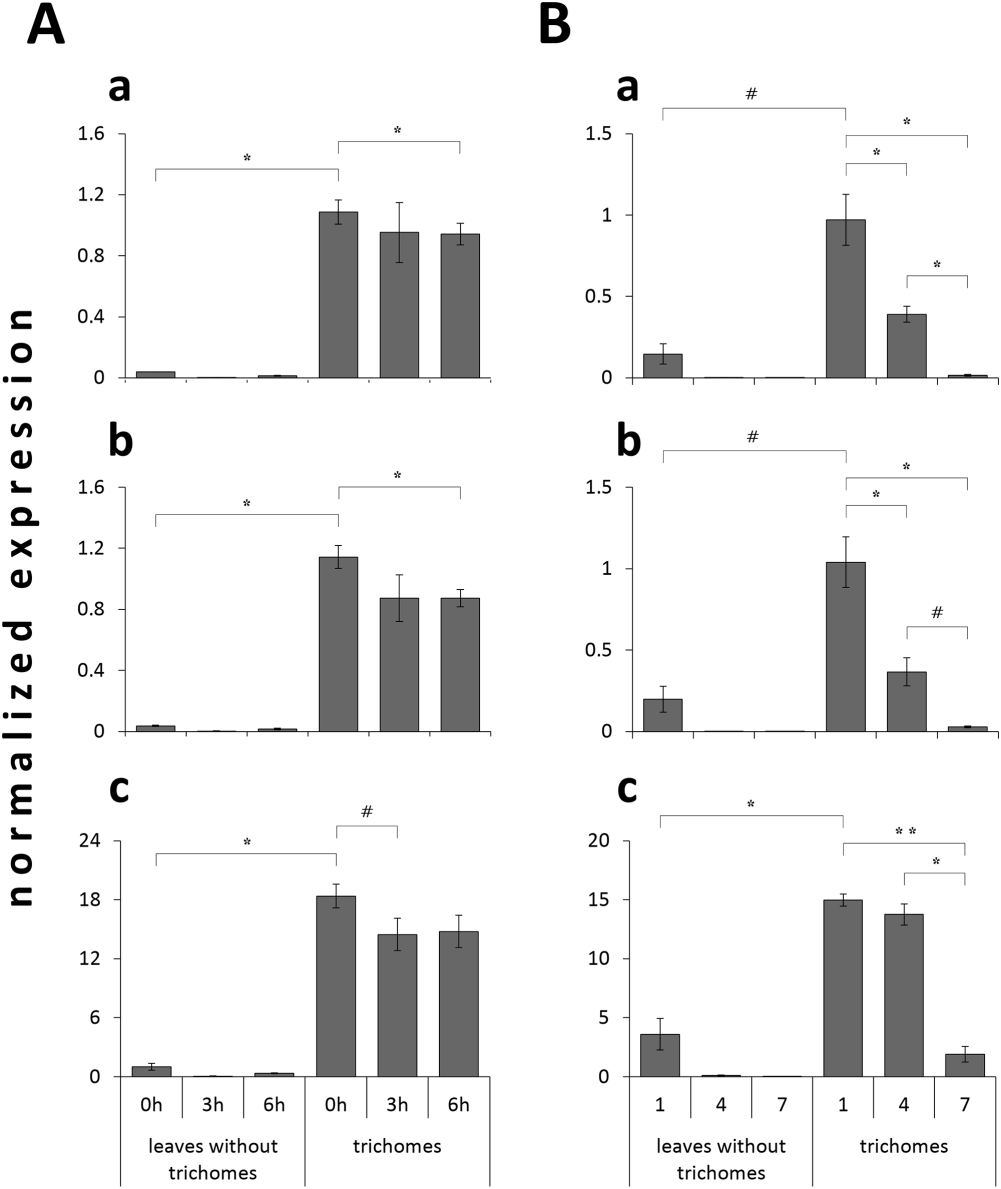
High-throughput qPCR analysis of *SfCPS* (a), *SfKSL* (b) and *SfFS* (c) genes in trichomes and leaves without trichomes performed on Biomark system. A) response of the genes 3 hours (3h) and 6 hours (6h) after mechanical wounding *in planta*. Zero hour (0h): non-wounded plants control. Data bars represent the mean expression levels from two biological replicates ± SE. B) expression profile of the genes in three different developmental stages of leaves (stages 1, 4 and 7, with 1 being the first expanded pair of leaves). Data bars are the mean values from three biological replicates ± SE. Transcript levels were normalized to *phosphatase 2A* (*PP2A*) gene (endogenous control). Asterisks and the hash symbol denote significant differences between two indicated values (^*#*^p < 0.075; *p < 0.05; **p < 0.01), based on Student’s *t*-test.

High-throughput quantitative expression analysis of isolated trichomes from different developmental stages of leaves and in response to wounding, as a simulation of insect feeding, revealed that the expression of *SfCPS* and *SfKSL* followed the same pattern with *SfFS* (Fig 9). Expression of these genes was highest in young leaves and dropped in old leaves, whilst it was suppressed by wounding after 3h or 6h.

### *SfFS*, *RoFS1* and *RoFS2* code for ferruginol synthases

**Fig 10:**
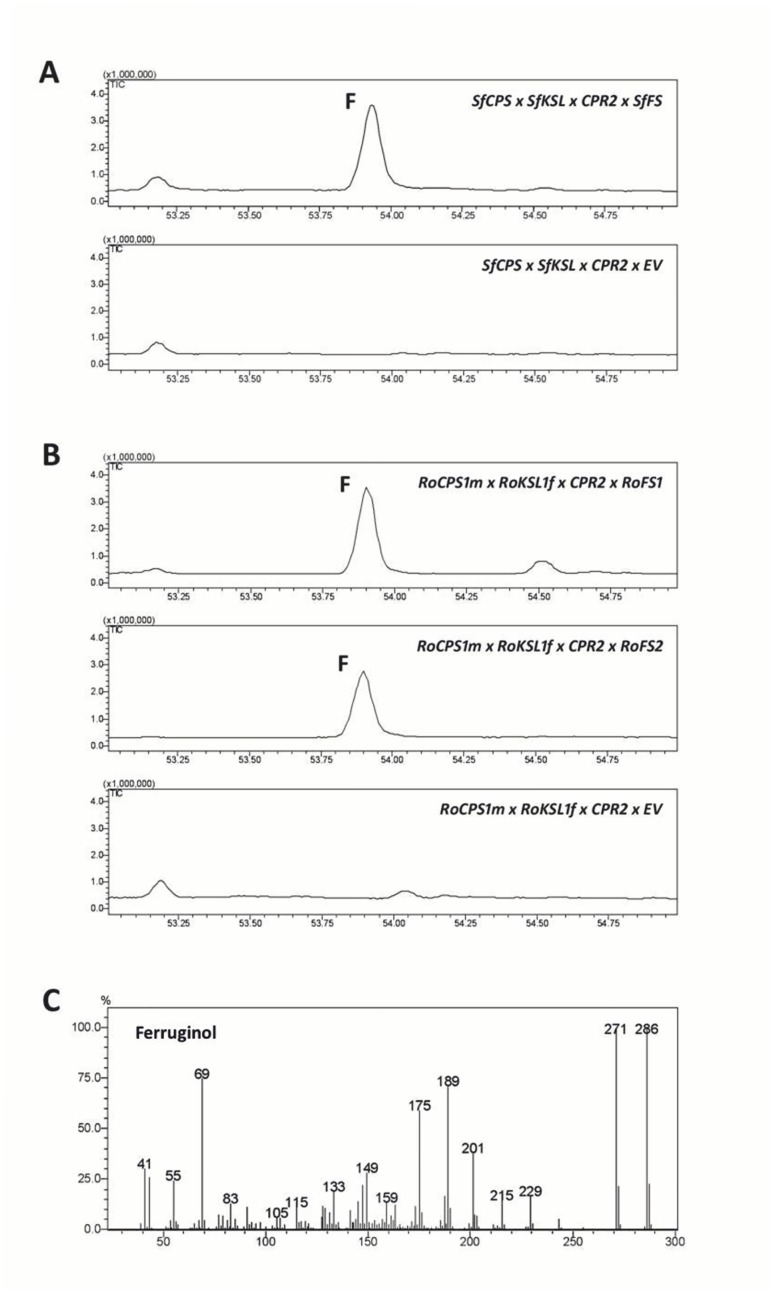
Heterologous expression of ferruginol synthase genes in yeast *(S*. *cerevisiae)*. A) GC-MS chromatograms of novel products secreted into culture media of yeast co-expressing *SfCPS*, *SfKSL* and either *SfFS* or the empty vector (EV) pWTDH3myc. B), GC-MS chromatograms of novel products secreted into culture media of yeast co-expressing *RoCPS1m*, *RoKSL1f* and either *RoFS1*, *RoFS2* and empty vector (EV) pWTDH3myc. F, peak corresponding to ferruginol. C), Mass spectrum of peak F.

The three candidate CYPs genes, presumably coding for ferruginol synthases from *S*. *fruticosa* (*SfFS*) and from *R*. *officinalis* (*RoFS1* and *RoFS2*) were expressed in yeast cells containing the two upstream diterpene synthase genes either from *S*. *fruticosa* or *R*. *officinalis*, as well as the cytochrome P450 reductase, *CPR2*, from Poplar [32]. Analysis of yeast extracts by GC-MS revealed the formation of ferruginol in all three strains analyzed (Fig 10). The identification of ferruginol was performed based on published mass spectrum [25].

**Fig 11:**
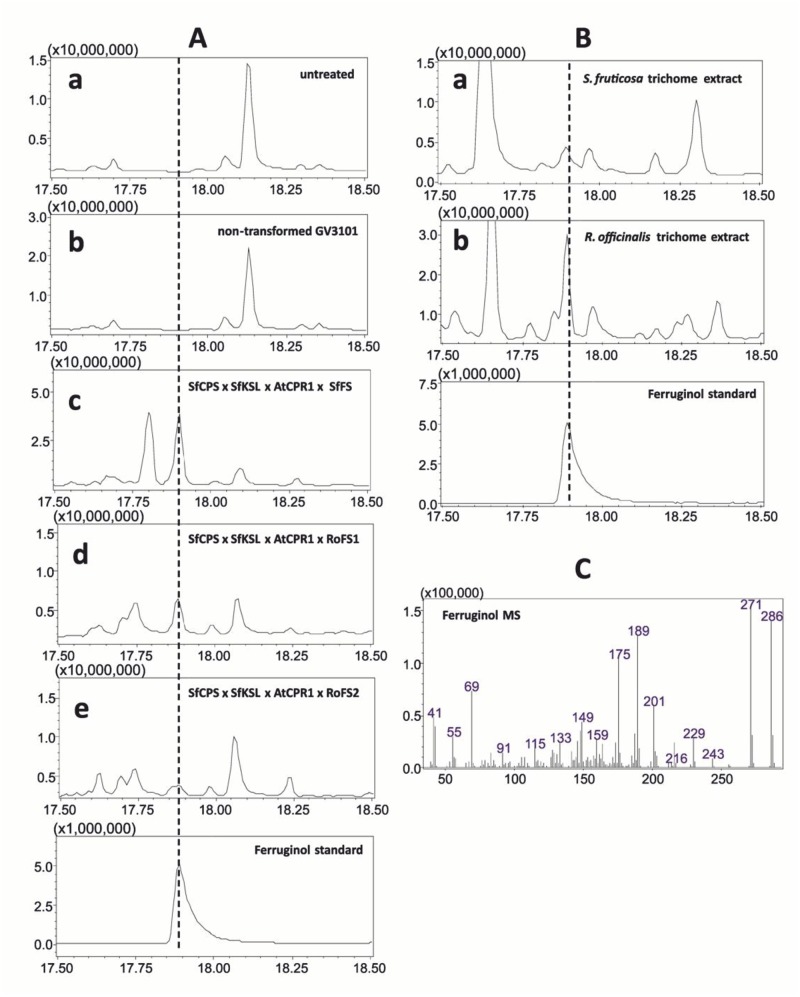
Production of ferruginol in leaves of *Nicotiana benthamiana* p19 transgenic plants. A) Total ion chromatograms of hexane extracts obtained from *N*. *benthamiana* leaves (a) untreated, (b) infiltrated with non-transformed *A*. *tumefaciens* strain GV3101, (c) infiltrated with *A*. *tumefaciens* strain GV3101 transformed with *SfCPS*, *SfKSL*, *AtCPR1*, *SfFS*, (d) infiltrated with *A*. *tumefaciens* strain GV3101 transformed with *SfCPS*, *SfKSL*, *AtCPR1*, *RoFS1*, (e) infiltrated with *A*. *tumefaciens* strain GV3101 transformed with *SfCPS*, *SfKSL*, *AtCPR1*, *RoFS2*. B) Ferruginol detection in hexane extracts of trichomes isolated from (a) *S*. *fruticosa* leaves and (b) *R*. *officinalis* leaves. C) Mass spectrum for ferruginol.

Further, all three putative ferruginol synthase genes mentioned above were subcloned in *Agrobacterium tumefaciens* binary vectors (*RoFS1* and *RoFS2* in pGA643 and *SfFS* in pART7/pART27) and *in vivo* functional characterization was performed through a transient expression in *N*. *benthamiana*. Ferruginol was detected in all transformed leaves’ hexane extracts when analysed by GC-MS confirming that these three genes, namely, S*f*FS, *RoFS1* and *RoFS2*, were coding for ferruginol synthases (Fig 11A). Chemical analysis of isolated trichomes from *S*. *fruticosa* and *R*. *officinalis* young leaves resulted in the detection of ferruginol (Fig 11B) showing the ability of young trichomes to synthesize this compound. In addition, this accumulation of ferruginol coincided with the expression of *SfFS* in trichomes of young leaves (Figs 9 and 11B).

## Discussion

In this work, we describe the isolation and characterization of the genes, namely *SfCPS*, *SfKSL* and *SfFS*, coding for enzymes involved in the biosynthesis of CA precursors in the young trichomes of *S*. *fruticosa*. These biosynthetic enzymes catalyse the formation of CDP, miltiradiene and ferruginol from GGDP, respectively. In addition, we functionally characterized two *R*. *officinalis* genes, *RoFS1* and *RoFS2*, homologous to *SfFS*, which catalyse the synthesis of ferruginol. Discovery of putative intermediates in the biosynthesis of CA brings us a step closer to fully understand the biosynthesis of this compound. Using this knowledge, currently practiced extraction from plant tissues could be replaced by more economical novel platforms for the biotechnological production of CA. Ferruginol, a probable precursor of CA, possesses many biological activities on its own [33–36], and the production of this compound using genetic engineering could be of great use for the pharmaceutical industry.

The abietane-type diterpenes, miltiradiene and ferruginol, were recently identified as precursors of tanshinones in *S*. *miltiorrhiza*, a Chinese medicinal herb [18,25] and putative precursors of CA in *R*. *officinalis* [24,26]. As in *S*. *miltiorrhiza* and *R*. *officinalis*, we confirm here that the synthesis of these abietane diterpenes involves two distinct terpene synthases and one CYP enzyme. The isolation of CYPs that synthesize ferruginol in *S*. *fruticosa* and *R*. *officinalis* was possible after a transcriptomic analysis of trichomes from young leaves of these plants (http://www.terpmed.eu/), a phylogenetic analysis with known plant CYPs (Fig 8) and gene expression correlation studies with *SfCPS* and *SfKS* (Fig 9). Subsequent expression of *SfFS*, *RoFS1* and *RoFS2* in yeast (Fig 10) and *N*. *benthamiana* (Fig 11) systems resulted in the production of ferruginol indicating that these genes are involved in the formation of ferruginol and ultimately in the synthesis of CA.

Phylogenetically, SfCPS and SfKSL show close relationship to other diterpene synthases responsible for the synthesis of secondary metabolites. In addition to CPS from *R*. *officinalis* and *S*. *miltiorrhiza*, in close proximity to the SfCPS amino acid sequence on the phylogenetic tree are also the enzymes involved in the biosynthesis of copal-8-ol diphosphate (labda-13-en-8-ol diphosphate) from *Cistus creticus* (CcCLS), *S*. *sclarea* (SsLPPS) and *Nicotiana tabacum* (NtCPS2) [20,37,38]. SfKSL shows high homology to miltiradiene synthase from *R*. *officinalis* (RoKSL1/2) and *S*. *miltiorrhiza* (SmKSL), as already mentioned, but also to SsSS and *Z*-abienol synthase from *N*. *tabacum* (NtABS) [37,38]. SfFS, RoFS1 and RoFS2 are closely phylogenetically related to the two ferruginol synthases already characterized from Lamiaceae family, namely CYP76AH1 (*S*. *miltiorrhiza*) [25] and CYP76AH4 (*R*. *officinalis*) [26]. All of these enzymes belong to the same single family or branch/subfamily, which is often the case with CYPs that metabolize similar or related compounds [31]. Interestingly, CYP76AH4 is only 87.8% and 85.6% identical to RoFS1 and to RoFS2, respectively (S12 Fig). This points to the existence of different isofunctional homologues of this enzyme in rosemary. It is also remarkable that a CYP from *S*. *fruticosa* reported here is more closely related to any of the *R*. *officinalis* CYPs (RoFS1, RoFS2 and CYP76AH4), than to the one from *S*. *miltiorrhiza* (CYP76AH1) (S12 Fig). The new functional homologues of ferruginol synthases retrieved in rosemary may represent, together with their homologue from *S*. *fruticosa*, new candidates for metabolic engineering and the production of a number of highly useful diterpenes. Their existence also reflects the diversity of cytochrome P450s and their evolution. Functionally redundant isoforms of proteins, often a result of gene duplication, may represent a base for the development of novel enzymatic functions. Cytochrome P450s playing redundant roles in plants have already been described [39,40]. More notably, this has been reported for some members of the CYP76 family, to which ferruginol synthases belong [41].

Finally, it is worth noting that SmKSL, RoKSL1/2 and *Salvia sclarea* sclareol synthase (SsSS) were recently described as rare examples of the bi-domain diterpene synthases belonging to the Tps-e/f subgroup lacking the γ-domain characteristic of the proteins [24,37,42]. The majority of plant diterpene synthase proteins of the Tps-e/f subgroup exhibit, on the other hand, the α/β/γ tri-domain structure. SfKSL provides a new example of a diterpene synthase exhibiting the bi-domain structure. As in the case of SmKSL, SsSS and RoKSL1/2, the homology of SfKSL to the other KSL sequences starts after their γ-domain, which indicates that the protein consists only of β and α domains, similarly to mono- and sesqui-TPSs.

Chemical analysis identified *S*. *fruticosa* genotype originating from Kavoussi as the one with the highest PDs concentration among the three genotypes investigated. This is in accordance with previous findings showing that the essential oil content of *S*. *fruticosa* plants changes along their native range on the island of Crete [43]. Kavoussi is a village located on the eastern side of the island, which has higher annual temperatures, higher annual sunshine and lower annual precipitation than the village Vrysses located on the western part of the island. It has previously been observed, by RAPD analysis of the *S*. *fruticosa* clones, that there is a genetic background to the variation of the chemical profiles, which most probably represents an adaptation to the varying local environments [44]. The genetic basis for the diversity of chemical profiles was afterwards confirmed by cultivating the clones originating from three *S*. *fruticosa* populations from different parts of Crete at the same location [45]. The clones, previously found to exhibit differences both in essential oil profile and total phenolic contents, maintained these differences under constant greenhouse and field conditions [45]. Therefore, the differences in PDs content shown in our work are genetic in origin and may present an adaptation to the environment from which these three genotypes come from.

It was also shown that the production of PDs was developmentally regulated with the young leaves having higher PDs content than the older ones. This pattern was also observed for CA content in *R*. *officinalis* [46] and for CA and C content in *S*. *officinalis* [47], as well as for labdane-type diterpenes from the leaves of another Mediterranean species, *C*. *creticus* [48]. High concentration of antioxidant PDs in the young, more vulnerable tissues, demonstrates the role of these compounds in overcoming various environmental stresses.

Lastly, our work revealed the trichomes as the site of ferruginol and CA accumulation in *S*. *fruticosa*. On the other hand, carnosol (C), a dehydrogenation product of CA, was predominantly detected in the leaves without trichomes. Whether C is synthesized from CA in the trichomes and then transported into the leaves, or that CA is transported into leaves and is then metabolised into C, remains to be elucidated. In any case, all the above information is critical for choosing specific tissues, developmental stages and conditions to apply functional genomic studies and especially deep RNA sequencing as a first approach for the isolation and characterization of genes participating in the downstream steps of CA and C biosynthesis. The results of chemical analysis were further verified by the higher expression of *SfCPS*, *SfKSL* and *SfFS* in isolated trichomes and in the early stages of the leaves’ development. Additionally, the expression of these genes was influenced by mechanical wounding. This reaction has so far been observed for many terpenoid biosynthetic genes of different plant species, and represents a defence strategy against invading organisms [49].

This study has elucidated the steps of the ferruginol biosynthesis and thus the likely initial steps in the synthesis of CA in *S*. *fruticosa*. It not only provides the basis for further investigation of the biosynthetic pathway of this valuable secondary metabolite, but it also adds to our knowledge in the field of terpene synthases research.

## Materials and Methods

### Plant material

*S*. *fruticosa* plants were grown either from seeds or explants collected from the wild populations in Crete, Greece (Kavoussi—Eastern part and Vrysses—Western part of Crete) or ordered from a commercial seed supplier (B & T World Seeds, Aigues-Vives, France). By decisions of the Forest Directorates of Chania (Western part of Crete) [decision 5482/24.7.13; https://diavgeia.gov.gr/doc/BΛ45OP1Θ-YP9] and Lasithi (Eastern part of Crete) [decision 4269/20.11.13; https://diavgeia.gov.gr/doc/BΛ10OP1Θ-O0Μ] collection of sage (*S*. *fruticosa*) is allowed under certain conditions: a) during the flowering-ripening season, using scissors or knife and without cutting all the stems of the plant in order to ensure its reproduction and b) maximum collected quantity is 500 gr. Following the above directions, we clearly state that no specific permissions were required for *S*. *fruticosa* sampling at the mentioned locations (Kavoussi—Eastern part and Vrysses-Western part of Crete). We also state that the field studies did not involve endangered or protected species.

Seeds purchased from the same supplier have represented the starting plant material for *Rosmarinus officinalis*. Plants were grown in the research greenhouse in the School of Agriculture, Aristotle University of Thessaloniki, Greece, under controlled temperature (25/18°C, day/night, winter, 32/20°C, day/night, summer) and natural photoperiod. The greenhouse had systems for climate control and data logger.

### Gene expression analyses

#### High-throughput quantitative expression analysis of *S*. *fruticosa* genes

For gene expression studies using the Biomark HD system, total *S*. *fruticosa* RNA was isolated from trichomes and leaves without trichomes from three different developmental stages of leaves (starting from the first expanded pair of leaves—stage 1, following with stage 4 and 7). Additionally, total RNA was prepared from trichomes and leaves without trichomes of mechanically wounded leaves belonging to the stage 1. Wounding was performed by cutting the leaves in uniform stripes with scissors *in planta*, and samples were collected after 0, 3, and 6 hours. Trichome isolation was performed using a combination of dry ice and synthetic brush abrasion methods, as explained in the Supplementary Information (S1 Method). Total RNA was isolated using Spectrum Plant Total RNA Kit (Sigma, USA) from three independent biological replicates for the gene expression studies of different developmental stages, while for the wounding assays, two biological replicates were used.

To remove traces of genomic DNA contamination, the RNA samples (1 μg) were treated with DNase I (DNA-free Kit, Ambion, USA) in a final reaction volume of 25 μl. After removal of genomic DNA, RNA quality was determined using Bioanalyzer 2100 (Agilent, USA). For RT-PCR, cDNA was synthesized from 400 ng of total RNA (DNA free) using Superscript III Reverse Transcriptase (Life Technologies, USA) and oligo-(dT) primers. Gene expression was quantified by real-time PCR using the Biomark HD instrument (Fluidigm, USA) and 2x SsoFast EvaGreen supermix with low Rox (Biorad, USA) dye. The cDNA samples were diluted to 6 ng/μl and pre-amplified using TaqMan PreAmp Master mix (Life Technologies, USA). Primers were used at a final concentration of 500 nM. After pre-amplification cDNAs were treated with Exonuclease I to remove leftover primers. The qPCR analysis was performed in technical duplicates following manufacturer’s instructions. Primer pairs for each gene candidate were designed based on the final isolated nucleotide sequences using Primer3 (http://bioinfo.ut.ee/primer3-0.4.0/) and PrimerQuest (http://eu.idtdna.com/PrimerQuest/Home/Index). The PCR efficiency for each primer pair used was calculated according to a dilution series from a pooled cDNA sample including all biological treatments. Expression fold was calculated using an efficiency-corrected delta-Ct method [50]. The *pp2a* gene was selected as endogenous reference for normalization of transcript levels.

#### qPCR of *S*. *fruticosa CPS* and *KSL* genes

For the analysis of gene expression, young (1–2 cm) and aged (3–4 cm) leaves were collected. Leaf material was frozen in liquid N_2_ immediately after the collection and stored at -80°C until further processing. Isolation of trichomes was performed using the dry-ice abrasion method (S1 Method).

High quality total RNA was isolated using the commercial Spectrum Plant Total RNA Kit (Sigma, USA), according to the manufacturers’ specifications with additional ethanol washes. cDNA was constructed from 1 μg total RΝΑ using the Superscript III kit (Life Technologies, USA) according to manufacturers’ specifications with 1 μg random primers (Life Technologies, USA).

For gene expression analysis, primer pairs were manually designed (S3 Table). The endogenous control selected and used for normalizing all quantitative real-time PCR (qPCR) analyses was the housekeeping gene elongation factor 4a (*elf4a*) [27]. Samples were prepared using the KAPA SYBR® FAST qPCR Kit (KAPA Biosystems, USA) and qPCR analysis for each sample was performed with three technical replicates in an Applied Biosystems 7500 Real-Time PCR System (Life Technologies, USA). General thermocycler conditions were 95°C for 2 min; then 6 cycles of 95°C for 35 s; 64°C for 30 s; 72°C for 30 s; followed by 35 cycles of 95°C for 20 s; 62°C for 20 s; 72°C for 15 s; a final extension at 72°C for 10 min and plate read at 76°C. To identify and verify the PCR products a melting curve was performed from 70°C to 95°C with readings every 0.1°C and a 10 s hold between observations. Relative expression values were calculated by the expression data of isolated trichomes relative to those from leaves without trichomes. qPCR data and the correct relative quantitation of results (RQ), and therefore the relative expression for genes, was calculated based on the efficiency-adjusted delta Ct method, where the fold change in the target gene was determined with the following formula:

Fold change = E-^ΔΔCT^, where ΔΔCT = (Ct_target gene_- Ct_Ref_) at Point X—(Ct_target gene_- Ct_Ref_) a Point Y, and E is the efficiency of the reaction [27].

It must be noted that each time, the statistical analysis takes into account the efficiencies of the reactions. Normalized expressions of the *SfCPS* and *SfKSL* genes were calculated using the software program Q-gene [51].

#### Isolation of *SfCPS* and *SfKSL* genes

For gene isolation, a leaf trichome cDNA library of *S*. *fruticosa* provided by previous work [27] was initially used. Two partial sequences of potential diterpene synthase genes (contig 66 and contig 195) were identified by sequence homology to known diterpene synthase coding genes. As a first step to obtaining the 5’ and 3’ region of the candidate genes, PCR amplification with Platinum Taq DNA Polymerase (Life Technologies, USA), was performed using the above cDNA library as a template. The PCR conditions were as follows: 1 cycle of 2 min at 94°C; 35 cycles of 30 sec at 94°C, 30 sec at 55°C, and 2 min and 30 sec at 72°C; and a final extension of 7 min at 72°C.

For contig 195 (putative *SfCPS* gene), combinations of gene-specific (793–03_F22_F1 and 04_K19_R) and library plasmid-specific primers (M13F and pDNRLib reverse 2) were used (S4 Table). In this way, additional but incomplete information about the *SfCPS* sequence was obtained, with the 5’ end of the gene still not revealed. To isolate the missing sequence of *SfCPS*, 5´ RACE System for Rapid Amplification of cDNA Ends (Life Technologies, USA) was further applied using *S*. *fruticosa* trichome total RNA as a template. Trichomes from young leaves were extracted using the dry-ice abrasion method (S1 Method). RNA extraction was performed using the Spectrum Plant Total RNA Kit (Sigma, USA). For the reverse transcription reaction, gene specific primer 5CopR172_GSP1 was used and the cDNA was (dC)-tailed. Amplification was performed using the two rounds of PCR, with a combination of the RACE kit primers (Abridged Anchor Primer—AAP, Abridged Universal Amplification Primer—AUAP) and gene specific primers (first round- 5CopR140_GSP2, nested- 5CopR83_GSP3) (S4 Table), using the described PCR conditions. The 5´ RACE has resulted in identification of the *SfCPS* complete sequence.

PCR amplification using the cDNA library did not result in isolating any additional sequence based on contig 66. It has resulted, though, in the isolation of 5’ and 3’ region of a similar gene, with even higher homology to the family of KSLs. The primers were 424_02_A10F and RCA02_A10_R (contig 66-specific), and M13F and pDNRLib reverse 2 (library plasmid-specific). Using the cDNA library, the sequence of this gene, named *SfKSL*, was fully identified. Finally, full-length cDNA of both *SfCPS* and *SfKSL* was amplified from trichome cDNA using Platinum Taq DNA Polymerase (Life Technologies, USA), ligated into pCRII-TOPO vector using the Invitrogen TA cloning system (Life Technologies, USA) and verified by sequencing. ORF sequences for both isolated genes have been deposited in the GenBank with the following accession numbers: KP091840 (*SfCPS*) and KP091841 (*SfKSL*).

#### Isolation of cytochrome P450 genes

The cDNA sequences coding for candidate P450s of *S*. *fruticosa* and *R*. *officinalis* were obtained based on trichome EST databases (http://www.terpmed.eu/). Two non-overlapping *S*. *fruticosa* singleton sequences (*Sfru*.*N01*.*C016156* and *Sfru*.*N01*.*C021415*), appeared to be transcripts of the same gene, according to a BLAST analysis. *Sfru*.*N01*.*C016156* sequence represented the 5′ end of the gene and contained the START codon, while *Sfru*.*N01*.*C021415* sequence comprised the 3′ region with the STOP codon. Primers for the full length sequence were designed based on the two singletons. For *R*. *officinalis*, two sequences with high similarity to each other (92,4% similarity of nucleotides), Roff.N01.C021640 and Roff.N01.C021643, were identified as candidates. The genes were amplified from the cDNA using Phusion Hot Start Flex DNA Polymerase (New England Biolabs, USA), cloned into pCRII-TOPO vector and sequenced.

#### Phylogenetic analysis

Multiple sequence alignments were generated using the ClustalW program. A Neighbor—Joining tree was constructed using MEGA version 5 [52] with a bootstrap of 1000 replicates, and rooted with *Physcomitrella patens* PpCPS/KS as an outgroup. Sequence analysis with TargetP 1.1 methodology has been used to predict the length of the transit peptide-coding sequences of the isolated genes (http://www.cbs.dtu.dk/services/TargetP/) [53]. A protein alignment using ClustalW (MegAlign, DNAStar) was done to calculate sequence distances in S12 Fig.

#### *E*. *coli* expression

Based on the predictions performed using the TargetP 1.1 algorithm, the region coding for putative transit peptide of *SfCPS* was 90 bp, and for the *SfKSL*, 147 bp long. Using these results, pseudomature versions (*m* in the construct name) of the two genes were cloned without the N-terminal transit peptide by PCR amplification from the pCRII-TOPO vector using Platinum Taq DNA Polymerase with the addition of restriction sites. PCR products were cloned into pCRII-TOPO vector. Upon sequence confirmation, the pseudomature fragments were subcloned into ptacHA expression vector, yielding the final plasmids ptacHA-*SfCPSm* and ptacHA*-KSLm*. ptacHA expression vector was created from pGEX-5X vector (Pharmacia, USA) by replacing a GST-tag with N-terminal polyhistidine (6xHis) tag and a Human influenza hemagglutinin (HA) tag.

The mature versions of SfCPS and SfKSL ligated into ptacHA vector were expressed in *E*. *coli* BL21-CodonPlus (DE3)-RIL competent cells. Liquid cultures of the bacteria harbouring the expression construct were grown to appropriate OD_600_ in Luria-Bertani medium supplemented with antibiotics for selection (50 μg/ml Ampicillin, 50 μg/ml Kanamycin, 17 μg/ml Chloramphenicol) with shaking at 37°C prior to induction with isopropyl thio-galactopyranoside (IPTG). After induction, cultures were grown at room temperature. In order to define the optimal conditions for bacterial expression of the mature SfCPS, combinations of different induction and growth parameters were tested (data not shown). Incubation at room temperature for 4 h of the culture induced with 0.4 mM IPTG, at the moment when the OD_600_ reached 0.5, gave the highest protein yields. These conditions were, thus, applied for producing the protein that was further purified and used in the enzymatic assays. For SfKSL, induction was performed in the same way as for SfCPS, with the only difference being that, after induction, cultures were incubated at room temperature for 16 h. Cell pellets collected after expression were either resuspended in SDS-PAGE sample buffer directly for SDS-PAGE analysis of crude extract, or stored at -20°C to be purified by Ni-NTA affinity chromatography.

Bacterial expression vector, ptacHA, carries an N-terminal polyhistidine (6xHis) tag, which has enabled the purification of overexpressed protein by Ni-NTA affinity chromatography. Cell pellets were resuspended in ice-cold lysis buffer (50 mM NaH_2_PO_4_, 300 mM NaCl, 10 mM Imidazole [pH 8.0]) with the addition of lysozyme (1 mg/ml) and 1 mM phenylmethylsulfonyl fluoride (PMSF). The amount of lysis buffer added was 1 ml per 10–20 ml of starting bacterial culture. The cell suspension was left on ice for 30 min, after which it was disrupted by sonication on ice until becoming slightly translucent. The lysate was cleared by centrifugation at 13000 g for 20 min at 4°C. 50 μl of 50% Talon metal affinity resin (Clontech, USA) equilibrated in lysis buffer was added per 1 ml of the cleared lysate. The bead suspension was incubated with rotation at 4°C for 60 min to allow the polyhistidine-tagged protein to bind the resin. The beads were then collected with centrifugation at 1400 rpm at 4°C in a benchtop cooled centrifuge. The beads were washed three times with washing buffer (50 mM NaH_2_PO_4_, 300 mM NaCl, 20 mM Imidazole [pH 8.0]). The amount of washing buffer was 1 ml per 100 μl of 50% Ni^2+^ agarose slurry. The polyhistidine-tagged protein was eluted by adding 100 μl of elution buffer (50 mM NaH_2_PO_4_, 300 mM NaCl, 250 mM Imidazole [pH 8.0]) per 100 μl of 50% Ni^2+^ agarose slurry. Three separate elution fractions were collected. Aliquots of the washing and elution fractions were analysed by SDS-PAGE, and the first elution fraction was stored in 50% glycerol at -80°C to be assayed for activity later.

Affinity purified SfCPS was assayed for activity by using 5 μl recombinant protein in a buffer containing 10 mM MOPS, pH 7.0, 20 mM MgCl_2_, 0.2 mM MnCl_2_, 1 mM dithiothreitol and 60 μM geranylgeranyl pyrophosphate (GGDP) as a substrate, in 0.25 ml final volume. Assay mixtures were incubated overnight at room temperature and the resulting mixtures were incubated at 37°C for 4 h with 0.025 units/μl of bacterial alkaline phosphatase (Takara, Japan) with the addition of equal volumes of hexane overlay. Hydrolyzed products were hexane extracted and traces of water were removed by adding a small amount of Na_2_SO_4_, which was next removed by centrifugation. The extracts were concentrated to 50 μl by rotary evaporation prior to analysis by GC-MS.

To identify SfKSL products, enzyme reactions contained 3 μl of SfKSL affinity purified protein in assay buffer, as above, containing 2 μl of purified SfCPS, 200 μM GGDP (substrate for SfCPS) in 50 μl final volume. Reaction mixtures were immediately carefully overlaid with equal volume of hexane, and left to incubate at room temperature overnight. Reaction products were hexane extracted and treated as above to remove traces of water. The extracts were stored at 4°C to be analysed by GC-MS.

#### Expression assays in *Saccharomyces cerevisiae*

*SfCPSm* and *SfKSLm* were both subcloned from ptacHA into yeast (*Saccharomyces cerevisiae*) expression vectors, pUTDH3 (P_TDH3_ 2 μ URA3) for the *SfCPSm* and pHTDH3myc (P_TDH3_ 2 μ HIS3) for *SfKSLm*, yielding the final plasmids pUTDH3-*SfCPSm* and pHTDH3-*SfKSLm*. The plasmids were co-transformed into AM104 yeast strain and the clones containing both plasmids were selected on glucose CM media lacking uracil and histidine. The final yeast strains were cultivated in 50 ml liquid media at 30°C and 250 rpm.

The strain AM104 was developed from the previously described AM102 strain [54], harboring heterozygous deletions in the genes *UBC7/ubc7*, *ssm4/SSM4*, *erg9/ERG9* and chromosomally integrated expression cassettes overexpressing a stabilized variant of *HMG2* was used the parental strain for further modification. The strain was modified with COD7/CcGGDPS1 cassette, which was developed by PCR amplifying the ORF of *CcGGDPS1* [29] using primers CcGGDPS1-BamHI and CcGGDPS1-XhoI (S5 Table). The amplified ORF was cloned into the COD7 integration cassette vector [54] (P_TDH3_-MCS-ts-LoxP-*HIS5*-LoxP) using BamHI and XhoI restriction enzymes generating the construct COD7/CcGGDPS1 (P_TDH3_-CcGGDPS1-ts-LoxP-*HIS5*-LoxP). The COD7/CcGGDPS1 cassette was then PCR amplified using primers 5-FLO8-COD7 and 3-FLO8-COD7 (S5 Table) which incorporate flanking sequences that are homologous to the 3’UTR region of *FLO8* gene. The cassette was transformed into AM102 cells and colonies growing in the absence of histidine were selected. The transformed colonies were tested by co-expressing *CrtI* and *CrtYB* genes of carotenoid biosynthesis. The strain with most intense coloration due to carotenoid production was selected. The *HIS5* selection marker was excised by transiently expressing Cre recombinase, as previously described [55], generating strain AM104 (S6 Table).

*SfFS*, *RoFS1* and *RoFS2* were subcloned from pCRII-TOPO constructs into the yeast expression vector pWTDH3 (P_TDH3_ 2 μ TRP1). *SfFS* was transformed into the above mentioned yeast strain AM104, which already contained the *SfCPSm* and *SfKSLm* gene constructs. *RoFS1* and *RoFS2* were individually transformed into the AM113 yeast strain containing the *RoCPS1m* and *RoKSL1f* gene constructs [24]. The AM113 strain harbors heterozygous deletions for *ERG9*, *UBC7*, *SSM4*, *MCT1*, *WHI2*, and expresses chromosomal inserted *GGDPS1* from *Cistus creticus*, the *FDPS1* from *Salvia fruticosa* and further contains two copies of a stabilized *HMG2* variant [24]. All the yeast strains were additionally transformed with a *PtCPR2* gene [32], cloned into the pYX143 vector. Produced volatiles by the transformed yeast cells were sampled by Solid Phase Micro-extraction (SPME) by using 2 cm-50/30 μm VB/Carboxen/PDMS StableFlex Fiber for Manual Sampling (Supelco, USA). Yeast cultures were grown in 50 ml flasks to saturation achieved after 24–48 h and volatiles were collected by SPME fibre for 30 min. The exposed SPME fibre was withdrawn into the outer septum-piercing needle and removed from the flask, which made it ready for analysis using GC-MS.

Liquid-liquid extraction of the 25 ml yeast cultures was performed with 3×25 ml of pure hexane. After overlaying with hexane, the cultures were sonicated for 15 min in an ultrasonic water bath at 40°C. Hexane extracts were combined, dried using anhydrous MgSO_4_, evaporated under a stream of air to a final volume of 100 μl and analysed by GC-MS. Extraction for the structural characterization by NMR was performed by overlaying yeast cultures (750 ml) with equal volumes of hexane followed by vigorous shaking by hand. The extraction was repeated three times.

#### Transient expression of *SfCPS*, *SfKSL*, *SfFS*, *RoFS1* and *RoFS2* in *Nicotiana benthamiana*

The entire open reading frame (ORF) of each gene (*SfCPS*, *SfKSL*, *SfFS* **,** *RoFS1* and *RoFS2*) was PCR-amplified from pCRII-TOPO constructs with the addition of restriction sites. *SfCPS*, *SfKSL*, *RoFS1 and RoFS2* sequences were subcloned into the expression vector pGA643 [56], while in the case of *SfFS*, pART7/pART27 binary vector system was used [57]. These expression vectors contain the CaMV 35S promoter and the *nptII* kanamycin resistance gene. Upon sequencing, constructs pGA643-*SfCPS*, pGA643-*SfKSL*, pART27-*SfFS*, pGA643-*RoFS1* and pGA643-*RoFS2* were introduced into *A*. *tumefaciens* GV3101 (pMP90) strain using the freeze-thaw method. An additional transformation was performed with cytochrome P450 reductase from *A*. *thaliana* (*AtCPR1*) cloned in pICH47732 vector. Transformed cells were grown on LB agar plates supplemented with antibiotics for selection of transformants until the appearance of visible colonies (rifampicin 50 μg/ml, gentamycin 25 μg/ml, and tetracycline 15 μg/ml for pGA643 constructs; rifampicin 50 μg/ml, gentamycin 25 μg/ml and spectinomycin 25 μg/ml for pART27 construct). LB broth containing appropriate antibiotics was inoculated from single colonies and the cultures were grown for 48 h at 28°C with vigorous shaking (250 rpm). Cells were harvested by centrifugation for 20 min at 4000×g. In addition, *Agrobacterium* strain harbouring a gene encoding the viral p19 silencing suppressor was also prepared [58]. For SfFS, RoFS1 and RoFS2 transient expression, *Nicotiana benthamiana* plants stably expressing the p19 silencing suppressor gene kindly provided by Dr. Kriton Kalantidis (Univ. of Crete, Heraklion, Greece) were used for the infiltration experiments.

Next, the cells were resuspended in the buffer containing 10 mM MES, 10 mM MgCl_2_ and 100 μM acetosyringone (Sigma, USA) to a final OD_600_ of approximately 0.5 and left to incubate at room temperature with gentle shaking (50 rpm) for 2 h. Equal volumes of *A*. *tumefaciens* cells from each gene construct resuspended in the buffer were used to infiltrate leaves of *N*. *benthamiana*, with a needleless 1 ml syringe. Plants were grown in the growth chamber at 23°C with 16 h light/8 h dark cycle for five days. After five days, infiltrated leaves were harvested and pulverized in liquid N_2_ with mortar and pestle. One gram of fine powder was extracted with 2×3 ml pure hexane after a brief vortexing followed by sonication for 15 min in an ultrasonic water bath. Combined hexane phases were separated from the precipitated leaf tissue after centrifugation for one min at 5000 rpm, dried using anhydrous MgSO_4_, evaporated under a stream of air to a final volume of 50 μl and analysed by GC-MS.

#### GC-MS analysis

GC-MS analyses for the *E*. *coli*, yeast and *N*. *benthamiana* extracts were carried out using a Shimadzu GC/MS-QP2010 system (Shimadzu, Germany) connected to a model QP-2010 electron impact (EI) mass spectrometer. Data acquisition and control of the GC/MS-QP2010 system was carried out using the GC-MS solution software. Chromatographic separation was achieved on an HP-5-MS capillary column (Agilent, USA) (30 m×0.25 mm inner diameter; 0.25 μm film thickness) for the yeast and *N*. *benthamiana* extract analysis, and a ZB5 (Phenomenex, USA) (0.25 mm×30 m, 0.25 μm film thickness) column for the *E*. *coli* expression extract. The carrier gas was ultra-high purity helium at a flow rate of 1.03 ml/min. For the enzymatic assay products (*E*. *coli* expression) the hexane extract was manually injected into the heated injector in the splitless mode. The inlet temperature was set to 230°C. The column temperature was raised from 60°C to 240°C with a rate of 3°C/min and maintained at this temperature for 5 min to equilibrate. For the yeast and *N*. *benthamiana* extract analyses, splitless mode was also used for the injector with the inlet temperature set to 230°C. The column temperature was initially held at 60°C for 1 min, then raised at 240°C with a rate of 3°C/min and held for 15 min. The extracts (1 μl) were injected automatically via the AOC20i-AOC20s autosampler. For the analysis of SPME sampled volatiles, manual injection of the device needle into the heated injection port of the gas chromatogram was performed.

Chromatographs in Fig 11 were generated using a 2010 Plus Shimadzu gas chromatograph with GCMS-QP2010 Ultra gas chromatograph mass spectrometer. Chromatographic separation was achieved on a MEGA-5 MS capillary column (MEGA s.n.c., Italy) (30 m×0.25 mm inner diameter; 0.25 μm film thickness). The column temperature was initially held at 50°C for 3 min, then raised at 300°C with a rate of 14°C/min and held for 3 min. The extracts (2 μl) were injected automatically via the AOC-20 Dual autosampler.

#### Structure elucidation

For structural characterization by NMR, yeast culture extracts were purified by silica gel flash column chromatography in hexane. This resulted in obtaining a fraction that contained 20 mg of the compound of interest, which was a satisfactory amount for the analysis by NMR.

NMR spectra for miltiradiene were recorded at 25°C on a Bruker Avance III at 300 MHz for ^1^H and 75 MHz for ^13^C, respectively, using CDCl_3_ as solvent. Chemical shifts are expressed in δ values (parts per million) relative to TMS as internal standard for ^1^H and relative to TMS (0.00 ppm) or to CDCl_3_ (77.05 ppm) for ^13^C NMR spectra. Coupling constants ^*n*^ *J* are reported in Hertz. Moreover, the complete assignment of ^1^H and ^13^C NMR signals was possible using 1D and 2D experiments (COSY H-H, HMQC, and HMBC) using the standard commercial pulse programs.

The first structure elucidation for miltiradiene was reported earlier by Gao et al. (2009) followed by Sugai et al. (2011) [18,59]. The mass spectrum of the product showed a molecular ion at *m/z* 272. The 20 different carbon signals from the ^13^C-NMR in conjunction with the molecular ion led to a possible diterpene structure without any symmetry. The four alkenyl carbon signals at 140.0, 135.5, 124.0, and at 116.5 ppm revealed two C = C double bonds. Further, 2D NMR data analysis suggested that the synthesized product was the miltiradiene. The proton signal at 1.26 ppm corresponding to the carbon resonated at 29.8 ppm is attributed to an impurity from hexane used for extractions. The complete assignment of proton and carbon chemical shifts is presented in S7 Table and in S4 Fig.

In rings A and B the methylene protons are arranged as axial or equatorial and resonated at about 1.1–1.3 and 1.5–1.7 ppm, respectively. The methylene carbons C7, C11 and C14 are attached to alkenyl carbons of ring C having an almost coplanar conformation. Therefore, the protons corresponding to these methylene groups are not diversified and give an almost identical pattern of multiplets. The almost identical methyl signals for C16 and C17 show two doublets with coupling of 7.0 Hz with the methine proton at C15. The differentiation of the two C4 methyl groups is achieved on the basis of NOE and ^13^C chemical shift data. Namely, there is a positive NOE between methyl protons resonated at 0.99 and at 0.86, corresponding to C20 and C19, respectively. There is also a significant difference in the ^13^C chemical shift data of the two C4 methyl groups attributed to their axial and equatorial configuration. C19 methyl and C20 methyl groups both being in axial configuration have 1,3-diaxial steric interaction resulting thus for both methyl carbons upfield resonances around 20 ppm, whereas the C18 methyl being in equatorial configuration resonates at 33.3 ppm.

The structure of miltiradiene, with numbering of skeleton as well as with assignments of proton and carbon NMR chemical shifts is depicted in S4 Fig. Moreover, in S5 Fig–S8 Fig the ^1^H and ^13^C NMR spectra are presented, whereas in S9, S10 and S11 Figs some diagnostic HMBC correlations between carbons and protons via ^*2*^ *J*_*CH*_ and ^*3*^ *J*_*CH*_ couplings are illustrated. In the case of cyclohexadiene ring C correlations are observed also via ^*4*^ *J*_*CH*_ couplings through the double bonds.

#### LC-PDA-MS analysis of phenolic diterpenes

Phenolic diterpenes were monitored in *S*. *fruticosa* a) whole leaves of all stages of development from the 3 different genotypes mentioned in “Plant Material” section, b) from young and old leaves of the genotype Kavoussi and c) from isolated trichomes and leaves without trichomes of very young leaves (appr. 1 cm long) of the genotype Kavoussi. The pots had 2 l capacity, containing peat and perlite in ratio 3:1. Plants were watered with a complete balance fertilizer every 20 days. The supplement light followed the natural photoperiod (10 h) and the distance between plants and lamps was 120–160 cm, with a light volume measured at 16–12 Klux. Trichome isolation was performed using the bead-beater abrasion method (S1 Method). The leaves' material was frozen in liquid N_2_ and ground to a fine powder with mortar and pestle. For each experiment, three independent biological samples were processed.

Next, compounds were extracted by adding 75% methanol containing 0.1% formic acid in a ratio of 5:1 (ml: g FW), followed by sonication for 15 min. Extracts were centrifuged and filtered over a 0.45 μm PTFE (Teflon) filter into an HPLC vial with glass insert. Phenolic diterpenes were separated, identified and quantified by using a LC-PDA-LTQ-Orbitrap FTMS system (Thermo Scientific) consisting of an Accela HPLC with photodiode array detector (240–600 nm) connected to an LTQ/Orbitrap hybrid mass spectrometer equipped with an ESI source. Injection volume was 5 μl. Chromatographic separation was on a reversed phase column (Luna C18/2, 3 μ, 2.0×150 mm; Phenomenex, USA) at 40°C, using a linear gradient from 5 to 75% acetonitrile (acidified with 0.1% formic acid) in 45 min and a flow rate of 0.19 ml/min. FTMS full scans (m/z 90–1200) were recorded in negative ionization mode with a resolution of 60,000. Phenolic diterpenes were identified based on similarity with the authentic standards of carnosic acid and carnosol, with respect to both absorbance spectrum, accurate mass, allowing a maximum of 3 ppm deviation from the calculated masses, and co-elution. Both compounds were quantified based on the chromatographic area of their specific mass.

#### Statistical analysis

Values are expressed as means ± SD or ± SE, and statistical significance was analyzed by Student’s t-test. All probability (p) values were based on two-tailed tests, and p < 0.05 was considered significant.

## Supporting Information

S1 Method Trichome isolation. (DOCX)

S1 Table Protein sequences used for phylogenetic analysis of *SfCPS* and *SfKSL* and their accession numbers. (DOCX)

S2 Table Protein sequences used for phylogenetic analysis of *SfFS*, RoFS1 and RoFS2 and their accession numbers. (DOCX)

S3 Table Primer sequences and combinations used in qPCR analysis of *SfCPS* and *SfKSL* and high-throughput quantitative expression analysis of *SfCPS*, *SfKSL* and *SfFS*. (DOCX)

S4 Table Primer sequences used for isolation and cloning of *SfCPS*, *SfKSL*, *SfFS*, RoFS1 and RoFS2. (DOCX)

S5 Table Primers used for the development of the AM104 yeast strain. (DOCX)

S6 Table Genotype of the strain AM104. (DOCX)

S7 Table NMR assignment of proton and carbon chemical shifts for the product synthesized. (DOCX)

S1 Fig Multiple amino acid sequence alignment for SfCPS, SsLPPS, SmCPS, NtCPS2, CcCLS and HvCPS. Black box indicates the aspartate-rich motif DxDD. N-terminal transit peptide sequence is underlined, and the predicted cleavage site is indicated by an arrow.
(DOCX)

S2 Fig Multiple amino acid sequence alignment for SfKSL, SmKSL, SsSS, ShSBS, SlPHS and NtABS. Black boxes indicate the aspartate-rich motif DDxxD. N-terminal transit peptide sequence is underlined, and the predicted cleavage site is indicated by an arrow.
(DOCX)

S3 Fig Multiple amino acid sequence alignment for SfFS, RoFS1, RoFS2, SmilCYP76AH1 and RoCYP76AH4. (DOCX)

S4 Fig Structure of miltiradiene with numbering of skeleton as well as with assignments of proton and carbon NMR chemical shifts. Shift numbers are rounded to one decimal digit for carbons and to two decimal digits for protons.
(DOCX)

S5 Fig ^1^H NMR of miltiradiene in whole scale. (DOCX)

S6 Fig ^1^H NMR of miltiradiene in aliphatic region. (DOCX)

S7 Fig ^13^C NMR of miltiradiene in whole scale. (DOCX)

S8 Fig ^13^C NMR of miltiradiene in aliphatic region. (DOCX)

S9 Fig HMQC 2D NMR of miltiradiene in whole scale. (DOCX)

S10 Fig HMQC 2D NMR of miltiradiene in aliphatic region. (DOCX)

S11 Fig HMBC 2D NMR of miltiradiene in whole scale. (DOCX)

S12 Fig Sequence distances between ferruginol synthases (% protein sequence identity). Calculated using a ClustalW protein alignment (MegAlign, DNAStar).
(DOCX)
