# Supplementary material for: Towards Elucidating Carnosic Acid Biosynthesis in Lamiaceae: Functional Characterization of the Three First Steps of the Pathway in Salvia fruticosa and Rosmarinus officinalis
Source: PLoS One. 2015 May 28;10(5):e0124106. doi: 10.1371/journal.pone.0124106 (PMC4447455; doi:10.1371/journal.pone.0124106)
Supplement: S1 Method — (DOCX) [file pone.0124106.s001.docx]

**Method S1. Trichome isolation**

*Dry-ice abrasion:* Glandular trichomes used as material for gene isolation and expression analysis were isolated by using the modified dry-ice abrasion method (Yerger et al., 1992). In short, excised leaves of *S. fruticosa* were cut in 0.5 cm strips and placed in a plastic 50 ml tube with screw cap and quickly frozen in liquid nitrogen. After removal from the liquid nitrogen tank, the tube was filled with approximately 3 cm^2^ of finely powdered dry ice to full volume. The tube was vortexed for approximately 2 minutes, after which the mixture was filtered using a sieve with a pore size of 1.6 mm to separate trichomes and dry ice from remaining leaf tissue. Immediately, pure trichomes (together with the dry ice) and remaining leaf tissue were placed in separate screw-capped tubes and returned to the liquid nitrogen tank, after which they were transferred to the -80°C freezer. The dry-ice method, although useful for preparing samples of pure trichomes, is not completely efficient in removing all the glandular trichomes from the leaf surface. For this reason, trichome-free leaves were prepared with the additional brush-abrasion step. Frost-resistant synthetic brush was used to clean the leaf surface from all remaining trichomes while constantly immersed in liquid nitrogen. In order to make sure that all trichomes were removed from both abaxial and adaxial sides of the leaves, final rinsing with ethanol (cooled in liquid nitrogen) was performed. All tissues were immediately kept at -80^o^C until further processed. For subsequent RNA extraction trichomes and leaves without trichomes were separately homogenized with liquid nitrogen using mortar and pestle.

*Glass bead abrasion:* For chemical analysis, the glandular trichomes were isolated using the modified Glass bead abrasion method (Gershenzon et al., 1992). Excised leaves were soaked in ice-cold, distilled water containing 0.05% Tween 20 for 1 h. After decanting the water the leaves were washed twice with ice-cold, distilled water, and abraded using a bead beater (Biospec Products, Bartlesville, USA). The chamber was ﬁlled with the plant material, 65 ml of glass beads (0.5 mm diameter), XAD-4 resin (1 g/g plant material), and ice-cold extraction buffer [25 mM HEPES pH 7.3, 12 mM KCl, 5 mM MgCl_2_, 0.5 mM K_2_HPO_4_, 0.1 mM Na_4_P_2_O_7_, 5mM DTT, 2.4 g l-1 sucrose, 26.4 g l-1 D-sorbitol, 6 g l-1 methyl cellulose, and 10 g l-1 polyvinylpyrrolidone (PVP; Mr 40,000)] to full volume. Following abrasion, the glands were separated from leaf material, glass beads, and XAD-4 resin by passing the supernatant of the chamber through a 250-µm test sieve. The residual plant material and beads were rinsed twice with 100 ml ice-cold isolation buffer (extraction buffer without methylcellulose) and passed again through the 250-µm sieve. The combined 250-µm ﬁltrates were then consecutively ﬁltered through 106-µm and 75-µm test sieves. Resulting filtrate was collected in several plastic centrifuge 50 ml tubes and centrifuged for 2.5 min at 2500 rpm. After this, the supernatant was decanted and pellet was transferred to a 1.5 ml reaction tube and stored in -80°C freezer. Remaining leaf tissue was separated from glass beads and XAD-4 resin by passing through a sieve with a pore size of 1.6 mm, transferred to the 50 ml tube and also stored in -80°C freezer.

The efficiency of trichome removal was confirmed by inspecting samples using a light microscope.
