# Supplementary material for: Towards Elucidating Carnosic Acid Biosynthesis in Lamiaceae: Functional Characterization of the Three First Steps of the Pathway in Salvia fruticosa and Rosmarinus officinalis
Source: PLoS One. 2015 May 28;10(5):e0124106. doi: 10.1371/journal.pone.0124106 (PMC4447455; doi:10.1371/journal.pone.0124106)
Supplement: S1 Table — (DOCX) [file pone.0124106.s002.docx]

**Table S1. Protein sequences used for phylogenetic analysis of *SfCPS* and *SfKSL* and their accession numbers.**

| Protein abbreviation | Species | Function | Accession number |
| --- | --- | --- | --- |
| AbCAS | *Abies balsamea* | cis-abienol synthase | AEL99953.1 |
| AgAS | *Abies grandis* | abietadiene synthase | Q38710.1 |
| AtCPS | *Arabidopsis thaliana* | ent-copalyl diphosphate synthase | NP_192187.1 |
| AtKS | *Arabidopsis thaliana* | ent-kaurene synthase | AAC39443.1 |
| CcCLS | *Cistus creticus* | copal-8-ol diphosphate synthase | ADJ93862.1 |
| CaCPS | *Coffea arabica* | ent-copalyl diphosphate synthase | ACQ99373.1 |
| CaKS | *Coffea arabica* | ent-kaurene synthase | ACQ99376.1 |
| CsCPS | *Croton sublyratus* | copalyl diphosphate synthase | BAA95612.1 |
| CsKS | *Cucumis sativus* | ent-kaurene synthase | BAB19275.1 |
| CmCPS1 | *Cucurbita maxima* | ent-copalyl diphosphate synthase 1 | AAD04293.1 |
| CmCPS2 | *Cucurbita maxima* | ent-copalyl diphosphate synthase 2 | AAD04292.1 |
| CmKS | *Cucurbita maxima* | ent-kaurene-synthase | Q39548.1 |
| GbLS | *Ginkgo biloba* | levopimaradiene synthase | AAL09965.1 |
| HaCPS | *Helianthus annuus* | copalyl diphosphate synthase | CBL42915.1 |
| HaKS | *Helianthus annuus* | kaurene synthase | CBL42917.1 |
| HvCPS | *Hordeum vulgare* | ent-copalyl diphosphate synthase | AAT49065.1 |
| HvKSL1 | *Hordeum vulgare* | ent-kaurene synthase | AAT49066.1 |
| JsCPS/KS | *Jungermannia subulata* | ent-kaurene synthase | BAJ39816.1 |
| NtCPS2 | *Nicotiana tabacum* | 8-hydroxy-copalyl diphosphate synthase | CCD33018.1 |
| NtABS | *Nicotiana tabacum* | cis-abienol synthase | CCD33019.1 |
| OsCPS | *Oryza sativa japonica* | ent-copalyl diphosphate synthase | NP_001046550.1 |
| OsKSL7 | *Oryza sativa japonica* | ent-cassa-12,15-diene synthase | Q00G37.2 |
| OsKS1 | *Oryza sativa japonica* | ent-kaurene synthase | BAE72099.1 |
| OsKSL10 | *Oryza sativa japonica* | Ent-sandaracopimaradiene synthase | Q2QQJ5.2 |
| OsKSL6 | *Oryza sativa japonica* | iso-kaurene synthase | ABH10733.1 |
| OsSynCPS | *Oryza sativa japonica* | Syn-copalyl diphosphate synthase | NP_001052171.1 |
| PpCPS/KS | *Physcomitrella patens* | ent-kaurene synthase | BAF61135.1 |
| PaIS | *Picea abies* | isopimaradiene synthase | AAS47690.2 |
| PaLAS | *Picea abies* | levopimaradieneabietadiene synthase | AAS47691.1 |
| PgCPS | *Picea glauca* | ent-copalyl diphosphate synthase | ADB55707.1 |
| PgKS | *Picea glauca* | ent-kaurene synthase | ACY25275.1 |
| PsiCPS | *Picea sitchensis* | ent-copalyl diphosphate synthase | ADB55709.1 |
| PsiKS | *Picea sitchensis* | ent-kaurene synthase | ADB55710.1 |
| PsCPS | *Pisum sativum* | ent-copalyl diphosphate synthase | O04408.1 |
| PtKS | *Populus trichocarpa* | ent-kaurene synthase | XP_002311286.1 |
| RcKS | *Ricinus communis* | ent-kaurene synthase (putative) | XP_002533694.1 |
| RcKSL1 | *Ricinus communis* | ent-kaurene synthase (putative) | XP_002525841.1 |
| RcKSL2 | *Ricinus communis* | ent-kaurene synthase (putative) | XP_002525836.1 |
| RcKSL3 | *Ricinus communis* | ent-kaurene synthase (putative) | XP_002525842.1 |
| SmCPS | *Salvia miltiorrhiza* | copalyl diphosphate synthase | ABV57835.1 |
| SmKSL | *Salvia miltiorrhiza* | kaurene synthase | ABV08817.1 |
| SsLPPS | *Salvia sclarea* | labd-13-en-8-ol diphosphate synthase | AFU61897.1 |
| SsSS | *Salvia sclarea* | sclareol synthase | AFU61898.1 |
| SdCPS | *Scoparia dulcis* | ent-copalyl diphosphate synthase | BAB03594.1 |
| SdKS | *Scoparia dulcis* | ent-kaurene synthase | AEF33360.1 |
| SmCPS/KS1 | *Selaginella moellendorffii* | labda-7,13E-dien-15-ol synthase | AEK75338.1 |
| ShSBS | *Solanum habrochaites* | santalene and bergamotene synthase | B8XA41.1 |
| SlCPS | *Solanum lycopersicum* | ent-copalyl diphosphate synthase | NP_001234008.1 |
| SlKS | *Solanum lycopersicum* | ent-kaurene synthase | AEP82778.1 |
| SlPHS | *Solanum lycopersicum* | phellandrene synthase | NP_001234629.1 |
| SrCPS | *Stevia rebaudiana* | ent-copalyl diphosphate synthase | AAB87091.1 |
| SrKS1 | *Stevia rebaudiana* | kaurene synthase | AAD3425.1 |
| SrKS2 | *Stevia rebaudiana* | kaurene synthase | AAD3424.1 |
| TcTS | *Taxus canadensis* | taxa-4(5),11(12)-diene synthase | AAR13860.1 |
| TaKSL5 | *Triticum aestivum* | nerolidol synthase | BAL41692.1 |
| VvKS1 | *Vitis vinifera* | ent-kaur-16-ene synthase (putative) | XP_002265005.2 |
| ZmTPS1 | *Zea mays* | acyclic sesquiterpene synthase | NP_001105097.1 |
| ZmCPS | *Zea mays* | ent-copalyl diphosphate synthase | NP_001105329.1 |
| ZmCPS2 | *Zea mays* | ent-copalyl diphosphate synthase 2 | AAT70083.1 |
