# Supplementary material for: Towards Elucidating Carnosic Acid Biosynthesis in Lamiaceae: Functional Characterization of the Three First Steps of the Pathway in Salvia fruticosa and Rosmarinus officinalis
Source: PLoS One. 2015 May 28;10(5):e0124106. doi: 10.1371/journal.pone.0124106 (PMC4447455; doi:10.1371/journal.pone.0124106)
Supplement: S2 Table — (DOCX) [file pone.0124106.s003.docx]

**Table S2. Protein sequences used for phylogenetic analysis of *SfFS*, *RoFS1* and *RoFS2* and their accession numbers.**

| Protein abbreviation | Species | Accession number |
| --- | --- | --- |
| AtCYP85A1 | *Arabidopsis thaliana* | AB035868 |
| AtCYP88A3 | *Arabidopsis thaliana* | AC000098 |
| AtCYP96A15 | *Arabidopsis thaliana* | AY090941 |
| CaCYP76B4 | *Camptotheca acuminata* | AES93118 |
| CrCYP76B6 | *Catharanthus roseus* | AJ251269 |
| GaCYP706B1 | *Gossypium arboreum* | AAK60517 |
| GmCYP71D9 | *Glycine max* | Y10490 |
| GmCYP93E1 | *Glycine max* | AB231332 |
| GuCYP72A154 | *Glycine max* | AB558153 |
| GuCYP88D6 | *Glycyrrhiza uralensis* | AB433179 |
| HtCYP76B1 | *Helianthus tuberosus* | Y09920,Y10098 |
| MpCYP71A32 | *Mentha x piperita* | Q947B7 |
| MpCYP71D13 | *Mentha x piperita* | AY281027 |
| MsCYP71D18 | *Mentha spicata* | AF124815 |
| MtCYP716A12 | *Medicago truncatula* | FN995112 |
| NtCYP71D16 | *Nicotiana tabacum* | AF166332 |
| NtCYP71D20 | *Nicotiana tabacum* | AF368376 |
| OsCYP76M7 | *Oryza sativa* | AK105913 |
| PaCYP71A1 | *Persea americana* | P24465 |
| PgCYP716A53v2 | *Panax ginseng* | JX036031 |
| PhCYP76A4 | *Petunia hybrida* | AB016061 |
| PsCYP720B4 | *Pisum sativum* | HM245403 |
| RoCYP76AH4 | *Rosemarinus officinalis* | DOI: 10.1039/c000000x/ |
| SaCYP76F37v1 | *Santalum album* | KC533717 |
| SaCYP76F38v1 | *Santalum album* | KC533715 |
| SaCYP76F39v1 | *Santalum album* | KC533716 |
| SbCYP51 | *Sorghum bicolor* | U74319 |
| SmCYP76B10 | *Swertia mussotii* | GU168041 |
| SmCYP76B4 | *Swertia mussotii* | D1MI46 |
| SmilCYP76AH1 | *Salvia miltiorrhiza* | AGN04215 |
| VvCYP716A15 | *Vitis vinifera* | AB619802 |
