# Supplementary material for: Towards Elucidating Carnosic Acid Biosynthesis in Lamiaceae: Functional Characterization of the Three First Steps of the Pathway in Salvia fruticosa and Rosmarinus officinalis
Source: PLoS One. 2015 May 28;10(5):e0124106. doi: 10.1371/journal.pone.0124106 (PMC4447455; doi:10.1371/journal.pone.0124106)
Supplement: S3 Table — (DOCX) [file pone.0124106.s004.docx]

**Table S3. Primer sequences and combinations used in qPCR analysis of *SfCPS* and *SfKSL* and high-throughput quantitative expression analysis of *SfCPS*, *SfKSL* and *SfFS*.**

| Primer name | Primer sequence (5’-3’) | Target gene | qPCR |
| --- | --- | --- | --- |
| For-eIF-4A-Sfru (Chatzopoulou et al. 2010) | TTGTTGCCATTGACATCTTCACTT | Elf4a  (acc. no FE536666) | Applied Biosystems 7500 |
| Rev-eIF-4A-Sfru (Chatzopoulou et sl. 2010) | CTCTCCCATGGCTGACATAACACT |  |  |
| SfruCPS-For2122 | ACCCTCACTAACAAAATATGCAAGC | CPS |  |
| SfruCPS-Rev2355 | AGCATCGGTGGTCTCATCATCG |  |  |
| SfruKSL-For1327 | TGGAGCGATGAGACGGAACTGAG | KSL |  |
| SfruKSL-Rev1558 | TATTTCCTGTATTTTCCTTGCGTTC |  |  |
| Sfru12591-PP2A-For1055 | AGGATGCAACAATCGAACAAC | PP2A | Fluidigm Biomark |
| Sfru12591-PP2A-Rev1287 | ACCCAACTGACTAGCAAGCAA |  |  |
| For2122-Sfru12304 | ACCCTCACTAACAAAATATGCAAGC | CPS |  |
| Rev2355-Sfru12304 | AGCACCGGTGGTCTCATCATCG |  |  |
| For1327-Sfru7690/1 | TGGAGCGATGAGACGGAACTGAG | KSL |  |
| Rev1558-Sfru7690 | TATTTCCTGTATTTTCCTTGCGTTC |  |  |
| Sfru16156-For268 | AAATACGGGCAGGTCTTCTCG | FS |  |
| Sfru16156-Rev418 | TGCTCTGGTGCGAGAACATC |  |  |
