# Supplementary material for: Towards Elucidating Carnosic Acid Biosynthesis in Lamiaceae: Functional Characterization of the Three First Steps of the Pathway in Salvia fruticosa and Rosmarinus officinalis
Source: PLoS One. 2015 May 28;10(5):e0124106. doi: 10.1371/journal.pone.0124106 (PMC4447455; doi:10.1371/journal.pone.0124106)
Supplement: S4 Table — (DOCX) [file pone.0124106.s005.docx]

**Table S4. Primer sequences used for isolation and cloning of *SfCPS*, *SfKSL*, *SfFS*, *RoFS1* and *RoFS2.***

| Primer name | Primer sequence (5’-3’) | Description | Gene name |
| --- | --- | --- | --- |
| AUAP | GGCCACGCGTCGACTAGTAC | 5’ RACE |  |
| AAP | GGCCACGCGTCGACTAGTACGGGIIGGGIIGGGIIG |  |  |
| M13F | GTAAAACGACGGCCAG | pDNRLib vector primers |  |
| pDNRLib reverse 2 | CTCCTAGGGAAACAGCTATGACCATGTTC |  |  |
| 793-03_F22_F1 | CAGCGCCTACCACGATGATGAGACCACCGAT | Library-PCR | *SfCDS* |
| 04_K19_R | cttgagttctatcaggttcaaatatggttgctg |  |  |
| 5CopR172_GSP1 | cggcgttctcgatgaatttcaagc | 5’ RACE |  |
| 5CopR140_GSP2 | tcatctttggtcttcatgaacacctgg |  |  |
| 5CopR83_GSP3 | gccatcgcctgactgcagtttcagaatc |  |  |
| SfCopS-5BamHI_0 – full length | GGATCCATGACCTCTATGTCCTCTC | Cloning |  |
| SfCopS-5BamHI_91 – truncated* | GGATCCGCATGCGCATGGCTGAACAG |  |  |
| SfCopS-3MfeI | CAATTGTCATACGACCGGTCCAAAGAG |  |  |
| 424_02_A10F | gatatatttttggaggttggtaaggct | Library-PCR | *SfKSL* |
| RCA02_A10_R | ccatcgctccatgtgtctaattctatcttg |  |  |
| 5SfKaurSfull(BamHI) – full length | GGATCCATGCTTCTTACCTTCAACATCACC | Cloning |  |
| SfKaurS_BamHI_147 – truncated | GGATCCACTCCTCCAACTGATTTG |  |  |
| SfKaurS_3_XhoI | CTCGAGCTATTCTCCAACGGGCTCATA |  |  |
| SfruCYP76n_EcoRI_F | GCATGAGAATTCATGGATCCCTTCCCTCTTGTAG | Cloning | *SfFS* |
| SfruCYP76n_XbaI_R | GCATGATCTAGATCACGCCTTAATCGGAACGATCTT |  |  |
| RoffCYP76nA_BamHI_F | GCATGAGGATCCATGGATTCTTTTCCTCTTCTC |  | *RoFS1* |
| RoffCYP76nA_XhoI_R | GCATGACTCGAGTTATACCTTAAACGGAACGATC |  |  |
| RoffCYP76nC_MfeI_F | GCATGACAATTGATGGATTCTTTTCCTCTTCTC |  | *RoFS2* |
| RoffCYP76nB_XhoI_R | GCATGACTCGAGTTATGCCTTAAAGGGAACTATC |  |  |

*truncated- ORF without the transit peptide
