# Supplementary material for: Towards Elucidating Carnosic Acid Biosynthesis in Lamiaceae: Functional Characterization of the Three First Steps of the Pathway in Salvia fruticosa and Rosmarinus officinalis
Source: PLoS One. 2015 May 28;10(5):e0124106. doi: 10.1371/journal.pone.0124106 (PMC4447455; doi:10.1371/journal.pone.0124106)
Supplement: S5 Table — (DOCX) [file pone.0124106.s006.docx]

**Table S5. Primers used for the development of the AM104 yeast strain.**

| **Primer name** | **Primer sequence (5’-3’)** |
| --- | --- |
| CcGGDPS1-BamHI | ggatccatgagaaggcgaatcgaacaaga |
| CcGGDPS1-XhoI | ctcgagctaattctgcctataagcaatgt |
| 5-FLO8-COD7 | aatttggggatgggtttaagccctgtgaactgaaccattttcacagcaacgactcagttcgagtttatcattatc |
| 3-FLO8 -COD7 | accattatgccaagctacttcaatgagtgtacatcaaccagaaaagtgcctgtggatctgatatcaccta |
