# Supplementary material for: Towards Elucidating Carnosic Acid Biosynthesis in Lamiaceae: Functional Characterization of the Three First Steps of the Pathway in Salvia fruticosa and Rosmarinus officinalis
Source: PLoS One. 2015 May 28;10(5):e0124106. doi: 10.1371/journal.pone.0124106 (PMC4447455; doi:10.1371/journal.pone.0124106)
Supplement: S6 Table — (DOCX) [file pone.0124106.s007.docx]

**Table S6. Genotype of the strain AM104.**

| **Strain** | **Genotype** |
| --- | --- |
| **AM104** | Mat a/alpha, P_GAL1_-(K6R)HMG2::HOX2, ura3,his3, trp1, P_TDH3_-(K6R) HMG2X2-::leu2, P_TDH3_-HMG2(K6R)::HO1, *ERG9/erg9,* UBC7/*ubc7, ssm4::/SSM4, P_TDH3_-CcGGDPS1-_3’UTR_ FLO8 derived from AM102* |
