# Supplementary material for: Towards Elucidating Carnosic Acid Biosynthesis in Lamiaceae: Functional Characterization of the Three First Steps of the Pathway in Salvia fruticosa and Rosmarinus officinalis
Source: PLoS One. 2015 May 28;10(5):e0124106. doi: 10.1371/journal.pone.0124106 (PMC4447455; doi:10.1371/journal.pone.0124106)
Supplement: S7 Table — (DOCX) [file pone.0124106.s008.docx]

**Table S7. NMR assignment of proton and carbon chemical shifts for the product synthesized.**

| Carbon  N^o^ | Chemical shift | |  |
| --- | --- | --- | --- |
|  | Carbon | Proton |  |
| 1 | 36.92 | 1.13 (dd, 13.2, 4.0 Hz, 1H, H_ax_), 1.66–1.76 (m, 1H, H_eq_) |  |
| 2 | 19.15 | 1.45–1.55 (m, 1H, H_ax_), 1.66–1.76 (m, 1H, H_eq_) |  |
| 3 | 41.96 | 1.13 (dd, 13.2, 4.0 Hz, 1H, H_ax_), 1.35–1.45 (dm, 13.2 Hz, 1H, H_eq_) |  |
| 4 | 33.43 |  |  |
| 5 | 51.73 | 1.17–1.24 (dm, 13.0 Hz, 1H) |  |
| 6 | 18.96 | 1.47–1.55 (m, 1H, H_ax_); 1.66–1.76 (m, 1H, H_eq_) |  |
| 7 | 31.91 | 1.88–2.04 (m, 2H) |  |
| 8 | 123.96 |  |  |
| 9 | 135.51 |  |  |
| 10 | 37.29 |  | x |
| 11 | 33.24 | 2.31–2.45 (m, 1H), 2.44–2.59 (m, 1H) |  |
| 12 | 116.54 | 5.445 (ddd, 5.4, 3.5, 1.8 Hz, 1H) |  |
| 13 | 140.02 |  |  |
| 14 | 25.53 | 2.58–2.67 (m, 2H) |  |
| 15 | 34.29 | 2.17 (sept, 7.0 Hz, 1H) |  |
| 16 | 21.20 | 1.001 (d, 7.0 Hz, 3H) |  |
| 17 | 21.33 | 1.016 (d, 7.0 Hz, 3H) |  |
| 18 | 33.32 | 0.893 (s, 3H) |  |
| 19 | 21.74 | 0.861 (s, 3H) |  |
| 20 | 19.64 | 0.993 (s, 3H) |  |
| s, singlet; d, doublet; m, unresolved multiplet; dm, doublet of multiplets, sept, septet | | | |
