# Supplementary material for: Towards Elucidating Carnosic Acid Biosynthesis in Lamiaceae: Functional Characterization of the Three First Steps of the Pathway in Salvia fruticosa and Rosmarinus officinalis
Source: PLoS One. 2015 May 28;10(5):e0124106. doi: 10.1371/journal.pone.0124106 (PMC4447455; doi:10.1371/journal.pone.0124106)
Supplement: S1 Fig — Black box indicates the aspartate-rich motif DxDD. N-terminal transit peptide sequence is underlined, and the predicted cleavage site is indicated by an arrow. (DOCX) [file pone.0124106.s009.docx]

**Figure S1**. **Multiple amino acid sequence alignment for SfCPS, SsLPPS, SmCPS, NtCPS2, CcCLS and HvCPS.** Black box indicates the aspartate-rich motif DxDD. N-terminal transit peptide sequence is underlined, and the predicted cleavage site is indicated by an arrow.
