# Supplementary material for: Towards Elucidating Carnosic Acid Biosynthesis in Lamiaceae: Functional Characterization of the Three First Steps of the Pathway in Salvia fruticosa and Rosmarinus officinalis
Source: PLoS One. 2015 May 28;10(5):e0124106. doi: 10.1371/journal.pone.0124106 (PMC4447455; doi:10.1371/journal.pone.0124106)
Supplement: S2 Fig — Black boxes indicate the aspartate-rich motif DDxxD. N-terminal transit peptide sequence is underlined, and the predicted cleavage site is indicated by an arrow. (DOCX) [file pone.0124106.s010.docx]

**Figure S2.** **Multiple amino acid sequence alignment for SfKSL, SmKSL, SsSS, ShSBS, SlPHS and NtABS.** Black boxes indicate the aspartate-rich motif DDxxD. N-terminal transit peptide sequence is underligned, and the predicted cleavage site is indicated by an arrow.
