# Supplementary material for: Towards Elucidating Carnosic Acid Biosynthesis in Lamiaceae: Functional Characterization of the Three First Steps of the Pathway in Salvia fruticosa and Rosmarinus officinalis
Source: PLoS One. 2015 May 28;10(5):e0124106. doi: 10.1371/journal.pone.0124106 (PMC4447455; doi:10.1371/journal.pone.0124106)
Supplement: S3 Fig — (DOCX) [file pone.0124106.s011.docx]

**Figure S3.** **Multiple amino acid sequence alignment for SfFS, RoFS1, RoFS2, SmilCYP76AH1 and RoCYP76AH4.**
