# Supplementary material for: Towards Elucidating Carnosic Acid Biosynthesis in Lamiaceae: Functional Characterization of the Three First Steps of the Pathway in Salvia fruticosa and Rosmarinus officinalis
Source: PLoS One. 2015 May 28;10(5):e0124106. doi: 10.1371/journal.pone.0124106 (PMC4447455; doi:10.1371/journal.pone.0124106)
Supplement: S4 Fig — Shift numbers are rounded to one decimal digit for carbons and to two decimal digits for protons. (DOCX) [file pone.0124106.s012.docx]

**Figure S4. Structure of miltiradiene with numbering of skeleton as well as with assignments of proton and carbon NMR chemical shifts.** Shift numbers are rounded to one decimal digit for carbons and to two decimal digits for protons.
