# Supplementary figures and images for: Towards Elucidating Carnosic Acid Biosynthesis in Lamiaceae: Functional Characterization of the Three First Steps of the Pathway in Salvia fruticosa and Rosmarinus officinalis
Source: PLoS One. 2015 May 28;10(5):e0124106. doi: 10.1371/journal.pone.0124106 (PMC4447455; doi:10.1371/journal.pone.0124106)

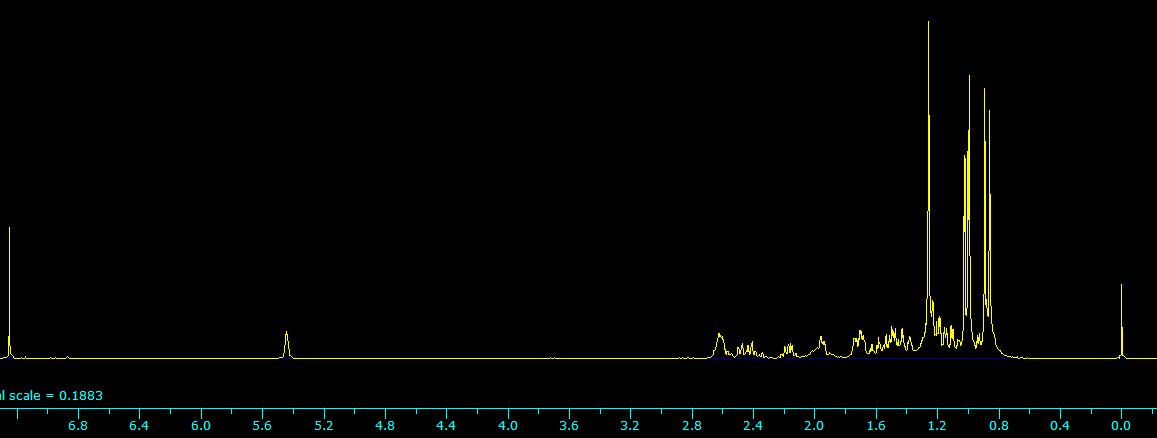


**Figure S5. 1H NMR of miltiradiene in whole scale.**

Supplement: S5 Fig — (DOCX) [file pone.0124106.s013.docx]

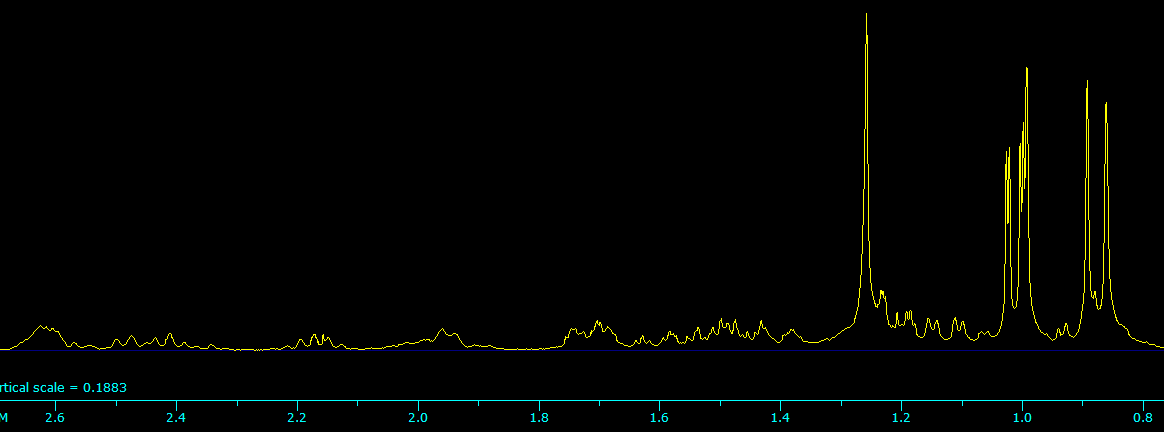


**Figure S6. 1H NMR of miltiradiene in aliphatic region.**

Supplement: S6 Fig — (DOCX) [file pone.0124106.s014.docx]

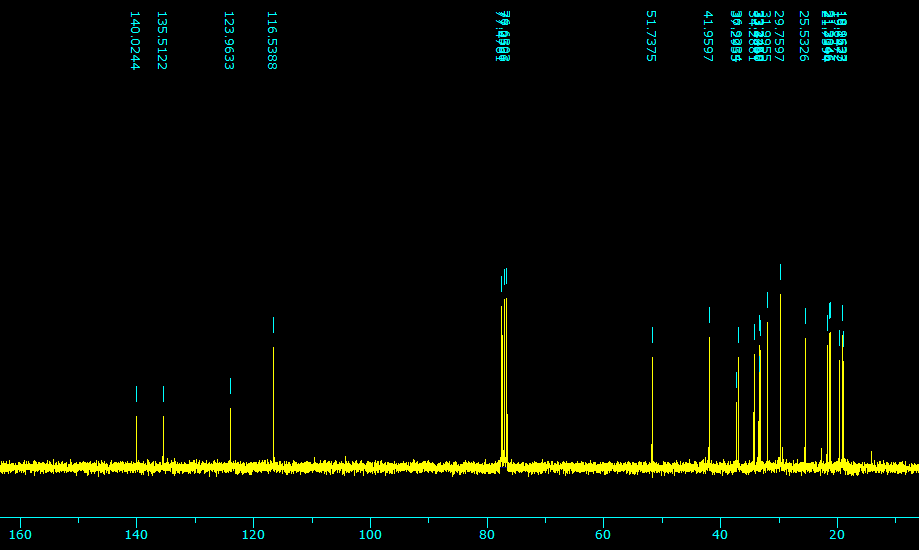


**Figure S7. 13C NMR of miltiradiene in whole scale.**

Supplement: S7 Fig — (DOCX) [file pone.0124106.s015.docx]

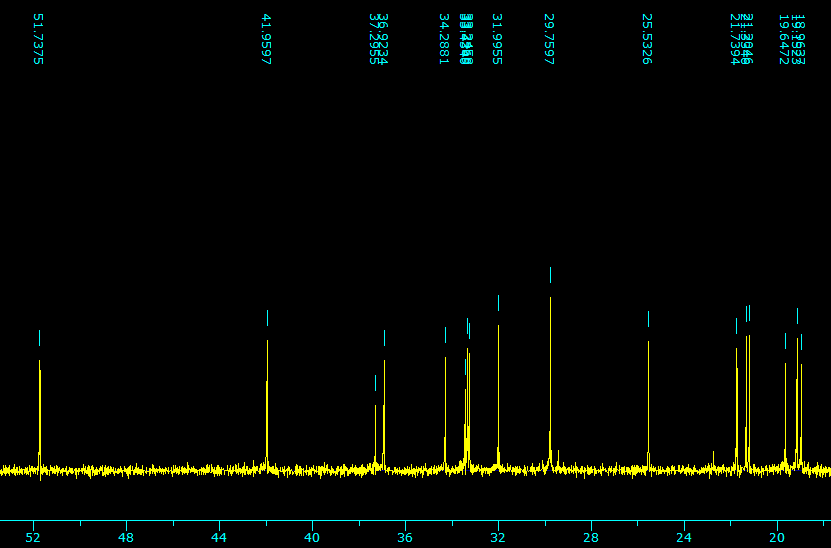


**Figure S8. 13C NMR of miltiradiene in aliphatic region.**

Supplement: S8 Fig — (DOCX) [file pone.0124106.s016.docx]

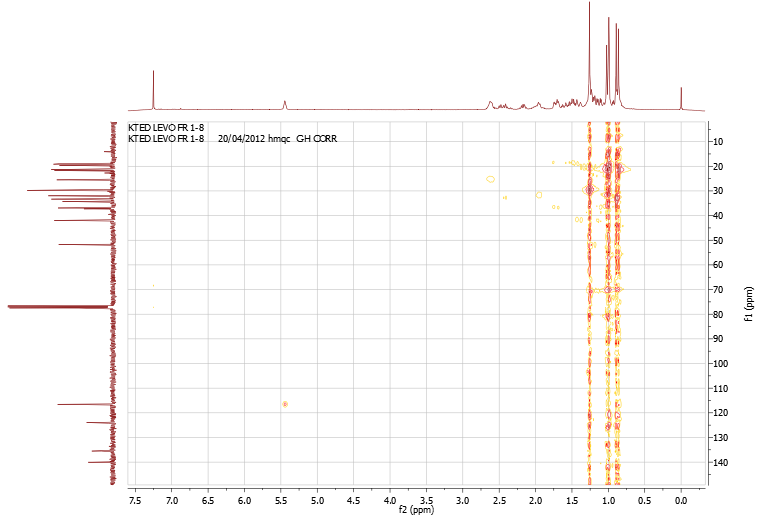


**Figure S9. HMQC2D NMR of miltiradiene in whole scale.**

Supplement: S9 Fig — (DOCX) [file pone.0124106.s017.docx]

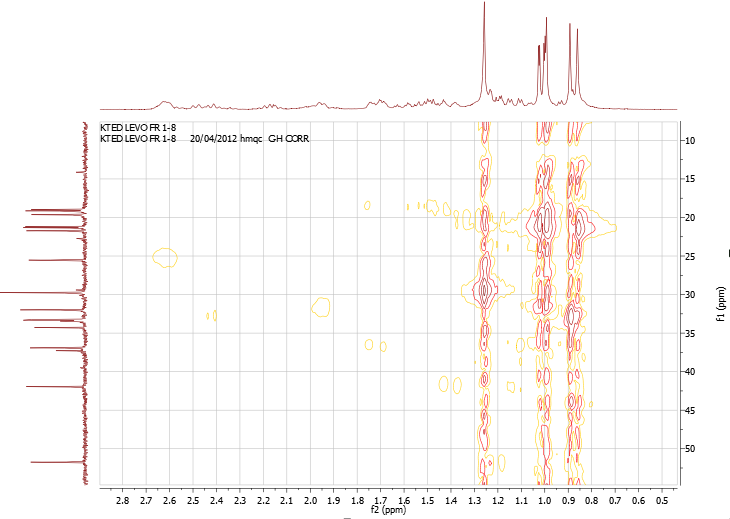


**Figure S10. HMQC2D NMR of miltiradiene in aliphatic region**

Supplement: S10 Fig — (DOCX) [file pone.0124106.s018.docx]

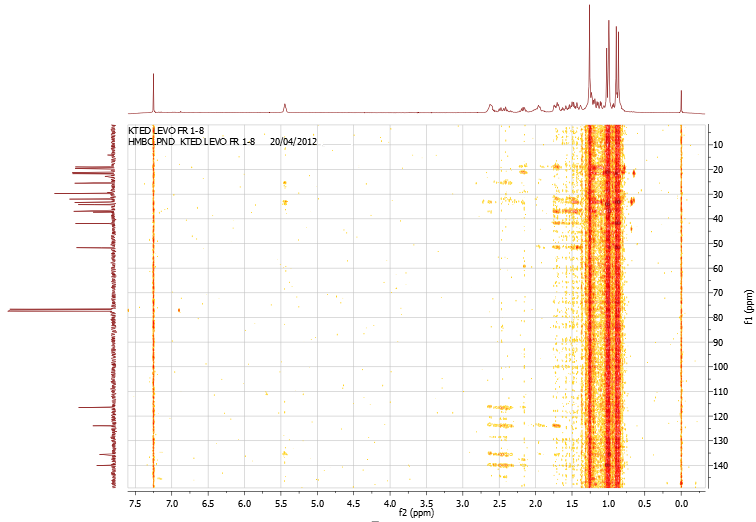


**Figure S11. HMBC2D NMR of miltiradiene in whole scale.**

Supplement: S11 Fig — (DOCX) [file pone.0124106.s019.docx]
