# Supplementary material for: Towards Elucidating Carnosic Acid Biosynthesis in Lamiaceae: Functional Characterization of the Three First Steps of the Pathway in Salvia fruticosa and Rosmarinus officinalis
Source: PLoS One. 2015 May 28;10(5):e0124106. doi: 10.1371/journal.pone.0124106 (PMC4447455; doi:10.1371/journal.pone.0124106)
Supplement: S12 Fig — Calculated using a ClustalW protein alignment (MegAlign, DNAStar). (DOCX) [file pone.0124106.s020.docx]

**
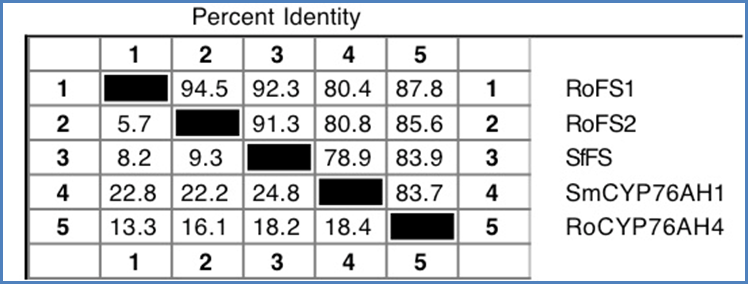
**

**Figure S12. Sequence distances between ferruginol synthases (% protein sequence identity).** Calculated using a ClustalW protein alignment (MegAlign, DNAStar).
